# Supplementary material for: Exploring the perspectives of healthcare workers and Program managers on the use of Truenat as a new tool for TB and DR-TB diagnosis in Nigeria: A qualitative study
Source: PLoS One. 2024 Dec 30;19(12):e0316204. doi: 10.1371/journal.pone.0316204 (PMC11684725; doi:10.1371/journal.pone.0316204)
Supplement: S1 File — (DOCX) [file pone.0316204.s001.docx]

**TRUENAT INTERVIEW TRANSCRIPTS**

**FGD_1**

Interviewer: Welcome great people to this Focus Group Discussion, Thank you for agreeing to participate. We are starting up at once just to gain time. I will start with participant number one for the first question. Thanks you for giving your consent to participate in the study and also for the permission to have the discussion recorded.

Question 1: P1, **Can you tell us about how a special day working with TRUNAT MTB RIF test for TB diagnosis goes for you**

**P1**: Thank you very much sir, the only thing I can say about that RIF testing is that since that December that they brought the machine to my facility I have not got any RIF patient through the machine but for diagnosis either negative or positive, RIF not detected, RIF Indeterminate and so on, are the results I have got for TB diagnosis but anything RIF patient I didn’t find such from the result.

Interviewer: Thank you. What preparatory steps do you take, how do you go about the test i.e your own personal experience

**P1**: I use to follow the SOP that was given to me in the facility so that I can get accurate result. But since then I didn’t have any RIF resistant present.

Interviewer: Number 2, can you tell us about how a typical day working with TRUENAT MTB RIF test for TB diagnosis goes for you

**P2**: Good evening, Generally, I will say that a typical day working in the Lab with TRUENAT MTB RIF Test for TB diagnosis has been encouraging and fine. It is less tedious, it is quite encouraging though on few occasions it was challenging, thank you.

Interviewer: what are 3 preparatory steps that are taken?

**P2**: Generally, first of all, after safeguarding myself and putting on…….gadgets then working on the sample collected, sputum sample. Most time, at the wash plate, you divide the sputum sample by adding small drops of insertion buffer to the sputum collected. Then smear and allow to stand for…… then after 10minutes, by then the sputum would have liquidified and being evacable, transfer about 0.5ml of potassium in the sputum with 1ml of pipette in elastic buffer bottle,. Then you add another 2 drops of potasssium buffer into the buffer bottle containing the sputum right now, then you smear and allow it for 3-5 minutes, then extract the……… and put into cartage. Then …into the machine,…by the time machine…work for about 20minutes by then it would have finished extracting the DNA sample that carries the TB. The machine will eject the cartage; you will now take the elute and drop into the eluence collection tube. From there you pick and drop in the sheet that you use to and launch into the TRUENAT machine, the analyzing machine. That will take about 40mls. All in all, we give about 1 hour to get the result of that sample. Thank you.

**P3:** Hello. Good afternoon. Working with TRUENAT machine has been a kind of experience one would like to have in addition to what one is doing before. So, I appreciate the provider and all our network people that we have been working with. Though working on a sample to detect for TB, I will say when we initially started with slide method that you need to stain, bring in visualized under microscope, the exposure is toward the sample but now with TRUENAT the exposure is a bit reduced. When you process your sample, you are sure there is going to be error free if you abide by the gold standard of the machine. If all things are being equal, you work with the gold standard, you are sure of the result you are ruling out, that is you are getting the result as accurate as it is from the patient. Working with the machine is quite loving though it takes time because of the time you have to devote to it alongside with our normal lab routine work. And now placing TRUENAT work which is the TB screening test is somehow cumbersome because of the duration of time involved most especially when you have a test coming out to be positive. You know you have to go extra mile spending over an hour to get your RIF done for that patient to know if patient is drug resistant patient or one that can go with Rifampcin-based treatment. So it has been cumbersome but it is quite enjoying to work with TRUENAT machine. Thank you.

Interviewer: What preparatory steps do you take, how do you go about the test i.e your own personal experience

**P3**: Like when the samples are being collected to the Lab. In our Lab we have …fridge where those samples are being kept, when you are ready and you put your samples on the bench to work, you make sure that your environment is tidy and same time you make sure you avoid error due to where you are not supposed to touch on those specimen. Like when I take pre-treatment stuff out for me to add my sputum, I make sure my hand do not touch the tip of pipette am going to use. Then when I want to open the lid, I make sure when am opening the lid because there is a particular lid inside the pre-treatment pack that is hard to remove, you have to see how to do that without contaminating the treatment, then I add my 0.5ml of the sputum, and add liquidified buffer of 2 drops you swell it on bench not shaking it and allow it to stay for 10mins until when you discovered that the liquidifaction is okay to pipette for you so as to avoid any form of clogging when you go for extraction, so when I put it into the cartage, before putting into the cartage when I have taken 0.5ml into pre-treatment stuff and I have added my liquidifying buffer of 0.2, after that add another 2 drops again and add it into the cartage, then from cartage into the machine for extraction then after extraction I go to the elute container and make sure I label it and to the PCR machine until the result comes out.

**P4**: Hello everyone. The TRUENAT is an additional experience that we have over the one we have before. It is very interesting. TRUENAT is very interesting platform for diagnosis of TB. I really enjoy it. It is quite interesting. The result is very reliable. I am sure of that.

Question 2: **How has your practice, TB testing, case finding and service delivery been affected by the introduction of new TRUENAT test machine**

**P3**: It has really helped us so much like I said before, the result is accurate, you are sure that what you have loaded in, if you avoided any form of error, you are going to get what you have put in. It has really been good working on TRUENAT machine. Finding TB case is very easy. You can work on so many samples on bench on same day and you time as to number of samples you want to do per day. If you able to stay with the machine, you can actualize it unlike before that you have to wait for drying. Working with TRUENAT machine has been so superb, wonderful and result being so accurate. Thanks.

**Question**: Has it affected TB case finding in your facility?

P3: Sure Truenat has improved TB case finding and turn-around time.

**Question**: Has it affected your work load?

P3: Am busier with TRUENAT. The number of samples that come in now has increased, combined with normal routine lab work that makes us busier.

**P2**: The TRUENAT practice has really been a smooth process…both in case finding and service delivery. Thank you.

**Question**: Has it affected TB case finding in your facility?

**P4**: It has affected in terms of turn-around time that we have. Even the sputum samples we are getting has doubled unlike before.

**Question**: Has it affected your work load?

Yes am very busy sir. Am spending a whole day in the facility performing TB diagnosis testing even during weekends. It makes us busier than before.

P6: Our workload increased both in testing and case finding. We are busier with the arrival of Truenat machine in our facility. We the Lab staff are always the last to leave the facility just to ensure that our results come out on time.

**Question 3**: **Practically, what has been your experience with use of TRUENAT machine compared to other TB testing platform that you have had experience with**

P8 Conducting maintenance is simple like changing the slide glass of the Truelab or flushing of the Trueprep. There is even a monthly maintenance log sheet that helps in maintenance.

**P7:** With Truenat, there is reduction in exposure to presumptive samples and tedious process of TB testing and assists me in multi-tasking unlike the hot Zieh-Neelsen method of TB testing that I have to run the whole process from start to the end.

**P3**: The TRUENAT machine, what I enjoy about it is that am sure and confident of the result am given, that it is going to be accurate, though the time come out of it sometimes when it coincides with my routine job. I have to abandon one for the other. What I enjoy about it is that even when you are not seated with the machine you can put in your samples to be running because you already have a time it going to stop. So you can also engage yourselves in doing some other things. You can leave and come back as at the time the processing time elapses. Unlike when we are working with smear for AFB. You have to finish the process of staining before you can say you want to opt out of where you are. After you have done your smear and drying, staining has to be done to the end before you leave. But with TRUENAT the moment I load it, am sure of getting my result within so so time.

Another thing I see about TRUENAT is that when we look at the result. It is going to also quantify it for you whether the result is low, medium or high. It is not that every patient is treated the same, it tells us the load in each patient body. In the aspect of smear for AFB we work with, we do that by kind of how we award what we see. I want to believe it is human judgement and because you have been told so so number of plus constitute scanty or many. When you take your slide for quality control, when they check it where you have scanty may turn out to be more than scanty and where you have assigned 7/100 may turn out to be more than that. With TRUENAT, there is no problem with grading. It tells you whether a patient is resistant to particular treatment/drug or not. When we were doing slides, there is nothing like quantifying whether one drug is good or not good. With TRUENAT in place it has made treatment of patient faster as it states whether patient is resistant or not. I enjoy working with TRUENAT than other medium of finding TB cases.

**Question**: How easy is it to do the test and how easy it to conduct maintenance?

**P3**: It is easy to do the testing and also the maintenance is also easy. Since we started working on the machine, I don’t think we had anything serious as a case that will lead to invite engineer to come and work on the machine. The machine has been working effectively. Maintaining it is not cumbersome.

**Question**: How is the through-put in terms of number of test and time for processing?

**P3**: The time for doing TRUENAT is a bit more because if you are getting a patient that is negative, you are thinking of spending like an hour and if you have RIF patient you will spend up to 2 hours. It is time consuming but it more advantageous.

**Question 3**: Practically, what has been your experience with use of TRUENAT machine compared to other TB testing platform that you have had experience with

**P4**: Comparing TRUENAT and GeneXpert test, all of them require qualitative sample for operation. Qualitative samples are samples that contain mucus not only saliva. This is what happens with TRUENAT, whenever you run a sample that do not contain mucus even if the patient is positive, it hard for the machine to show you that it is positive. Whenever the sample contains mucoid, you automatically get a reliable result.

**P4**: The TRUENAT test is easy to conduct when you follow the SOP. Though the GeneXpert is easier.

**P4**: The maintenance is so easy when you know it and difficult when you don’t know it. When you are maintaining, you are required to switch it off because of screen you can touch where you are not supposed to touch. It does not require pressure because it is fragile. When you are running a test it will shock you the number of test that is remaining. When you do not do flushing very well the machine will not provide the elute for you. It is also required that after doing 50 tests you change the reagent.

Question 4, **In the field, in combination with PDX card to support testing for community acute case finding / have you had that experience**?

P4 : yes , what is going well is that in the field, you are going to prepare your case well and you are just doing your job and what does not go well with the community outreaches is that people see it as something magical especially our people in the local communities, they are seeing the Truenat machine as something this is small and cannot imagine how such a small thing could be able to diagnose TB. They say we are magicians. This is the kind of challenge you see.

The machine when brought out from the box to perform test, during harmattan season we experience some kind of dust and something like that.

When I started using Truenat machine, some DOTS providers did not accept the Truenat result because it is something new to them, maybe some were not aware or don’t have any knowledge of it so we print something like POS result and give them and they didn’t accept it unless when we stay and show them the result on the screen for them and they see that it is the result for the screening they now believe that the result is reliable and can accept it so those are among the thing that didn’t go well in the field .

P3; On two occasions I have been able to work outside the facility with the Truenat machine to do community service for case finding for TB and the experience I see in it is that you will be able to streamline your presumptives with the help of the x-ray. In our last community outreach, we had 12 presumative cases from almost 100 people that came for the screening exercise. Out of the 12 that were tested, three were positive for TB. So this approach helps us to be able to go straight in getting the patients on time without you having to work on some many samples before you can actually get one to be positive. You will be able to have your result there at the field because Truenate machine has an inbuilt battery. You don’t need electricity to work on it, so when you run the test, you get your results and because the DOTS people are on ground, the patients are placed on treatment immediately and if there is a need for the team to do contact tracing that will be done. That is what I see about the outreach.

QUE: any bad experience from such acute case finding in the community

P3: I wouldn’t say that they have been any bad experience especially from the outreach I have been so far, in terms of patients coming we have enough kits to work with and you have time within your constrain that you can still work with and still get your result on time and give to the patient, in-fact I enjoyed doing because when you see people coming around to be tested you are happy that people are able to come around and participate and know their health status. So I don’t see anything bad in it.

**QUES: Tell us the major advantage and major disadvantage with the use of TRUENAT machine for TB testing?**

**P7:** Generally, Tuenat is user friendly, if you can operate an android gadget you can operate Truenat. The result from Truenat test is accurate, precise and reliable. Also, it detects RIF Resistance. Its major drawback is that it cannot be used for follow up samples since it detects both dead and living bacilli.

**P6** Truenat is easy to operate. Maintenance processes like flushing and changing of slider glass are easy to perform. The machine could be charged when there is power and used when there is no available power.

The disadvantage is that in a typical day, we perform 8-12 tests. This number is not encouraging when compared to the workload in a day which could be between 50 to 100 samples

P3: The only thing I see about it is that Truenat is accurate even when we have a school of thought that still prefer GeneXpert to Truenat but I still see that Truenat is ok but the only thing I see about Truenat is that Truenat is sensitive, so if you mistakenly touch your hand on any of the chip surface or you allow your pipette to touch the chip by breaking a kind of slip inside you are not likely to get your result so because of that I see it to be more sensitive but accurate.

QUEES: any major disadvantage

P3: I have not been able to think of anything too major on the aspect of Truenat to be called a disadvantage.

QUES: but the main fact that we do not use stool to test for TB using Truenat, do you see it as a disadvantage?

P3: No, I don’t really see it as a challenge because we are made to understand that it is still work in progress. There are many tests we could conduct using Truenat, it is just that they need to update it then we can start testing for TB using stool.

QUES: what about the main fact that you load only two samples, you still did not see it as a disadvantage?

P3: since this is what we have to work with, like I have been working with slide for sometime now so we adjust to it. It is cumbersome working with the two bays where you have so many samples on ground, but since that is what we have on ground now, we should adjust ourselves to it. I expect that there will be other Truenat machines with higher number of modules so we make do with what we have on ground pending when it will be upgraded. I have never work with GeneXpert but I learnt it has many modules for you to work with.

**QUES:P4 can you tell us the advantage and major disadvantage with the use of Truenat for TB testing?**

P4: The major advantage is that the Truenat machine is very sensitive and very simple to use to diagnose TB cases, moreover it is very simple to carry about because it has battery and when you recharge it, it can be used for a long time to test for TB when there is no light.

On the other hand I have not seen any major disadvantage in using Truenat to test for TB. However, sometimes the machine gets hot when you work with it for a long time in the field, I have noticed that when it gets hot it starts giving invalid results. That is the major disadvantage that I have experienced with Truenat. When you lose your elute, you will go and find another sample to extract and obtain another elute that you are going to use and prepare your test, so whenever you are running a test and it says invalid you have to know that the invalid is from the elute, majority of the invalid is not from the Truenat machine but from the elute, so you have to go and find a fresh sample and start another procedure to obtain your elute.

QUESTION 6: **P3 can you tell us about the challenges you have encounter using Truenat machine at different component?**

P7: If only one can load more than one cartridge on the TruePrep it would be better. Also, the rechargeable battery does not last up to 8 hours. This makes it necessary that the Truenat machine should have solar power supply.

P:3: The challenge I have encountered is that Truenat machine is highly sensitive and because of the sensitivity of Truenat machine there are some samples that it finds difficult to run, like when you have a bloody sample, the test will be repeated. When we have been told not to work on a bloody sample but there are some samples that you might not actually notice that they are blood inside even when the extraction is completed and you get a good sample to extract, you will discover that it come out to be clogged and most of the time when it clog like that, for you to just tell the patient to produce another sample, you will find that it is going to take some time before you can get the patient, you know from you to the person that brought the patient to the lab, because you don’t want to miss out on anything of the patients so you have to go back to the previous way of doing it which is the SLIGHT METHOD, this is what we do majorly in my facility, you just make sure you go and do what we call ANP so that you will be able to get result for the patient and we discover that unless a patient

QUUESTION: P4 can you tell us about the challenges you encountered using Truenat machine?

P4: I have noticed that when there is no steady electricity, although the battery do last but you will not perform very well on time unless you find a way to re-charge the equipment, secondly the sample need to be qualitative sample, a qualitative sample in one that is mucoid (some people just produce ordinary saliva). Whenever the sample is not qualitative the result will automatically be negative, even if the patient has clinical signs for TB vocation………………… most of the dot provider does not accept Truenat result until when they give them the paper, they didn’t accept the result as the diagnose result I have asked my boss MR………… how is this thing going to be resolve so that people will understand that this is the new implementation of TB diagnosing platform and he asked me that we have to print the result, when we transfer the result from the machine we share it through the Bluetooth and send to our phone and print the result and is interpreted to them, so these are the challenges we have been facing for diagnosing of TB through Truenat.

QUESTION: If they ask you now, what is the commonest challenge with the use of Truenat what will you say?

P4: Commonest challenge is printing the result because for me now in my facility I have stopped using Truenat printer to print the result. I only print the Truenat test result using the normal printer that we know. I have to send the result to my phone and then print it so that the DOTS providers and other health workers will accept the Truenat result as reliable and true diagnosis for TB. This is commonest challenge I have encountered.

QUESTION: Are you saying that they don’t accept the printed result?

P4: yes, most of them are not accepting the result from the Truenat printer, they are seeing it as something like POS. Giving them something like POS. This is why I have stopped using Truenat printer for print the result so I have now using normal printer for printing and photocopying for the result.

QUESTION: Does that mean that all the challenges am seeing does not affect the down time?

P4: what do you mean by down town sir?

QUESTION: that it does not affect your output, you continue your work, the only thing is that once you print your result through Truenat printer, it doesn’t appear genuine or authentic ABI?

P4: yes, they are saying that it is not an authentic result but that doesn’t affect my work sir because I know what am doing and I trust what am doing so that does not stop my work or affect my work because it is something that requires knowledge and science to do so this does not affect my work and thank GOD now We have started enlightening people and 60% of the people are now accepting our Truenat results, this story I am telling happened about 3-5 months ago that was when we faced this kind of challenge but now the result is becoming acceptable to the people.

**QUESTION: P4 what have been your experience with the Truenat service providers since implementation, have they been up and doing? [Service providers are peoples who come to repair the machine any time it spoils]**

P4: Yes they are coming.

QUESTION: How do you see the turnaround time of the Truenat?

P4: The turnaround time is very good because when we receive the samples, the highest turnaround my facility have given is 48 hours. I do whatever I have to do and not allow the samples to exceed 48 hours in my hand without processing it. This is what makes me work even on holidays and even weekends, I have come to conduct my tests so as to get our report in a very good turn-around time.

QUESTION: P3 what have been your experience with Truenat service report providers since implementation?

P3: So far, the Truenat service providers have been up and doing since I started using Truenat machine though we have not had a cause to call them for repairs but when it comes to coming around to maintain the machine, they have always been coming around either to upgrade or update the Truenat machine and the likes. I will say that the maintenance team are doing wonderful...

QUESTION: P3 How do you see the turn-around time of the Truenat?

P3: The turn-around time is fine although it is on a high side, it would have be more preferred if the turn AROUND time is not as long as it is BEING programmed because with that we can be able to attend to so many samples per day and then you will be able to cover a lot.

QUESTION 8: P4 What have been your experience with supervisory/mentorship visit since implementation of TRUENAT?

P4: I have experienced a lot of courage and confidence working with Truenat machines, whenever they come for supervision they encourage me to stick to the work and that this is not the work for lazy people, it requires knowledge and competence and the result is automated and even the machine is bringing out reliable result, so I have experienced a lot of courage from my supervisory people.

P3: It has been encouraging when they come about for supervising and checking of what we do because you have to know that what you are doing is okay because you have some people that will come around or maybe even those that are seeing your record from the back view because we learnt that the device is connected beyond our facility so we are also making sure that they put more effort so that this reduce our number of invalid as much as possible and our number of errors.

**Question 8. What has been your experience with supervisory/mentorship visit since implementation of TRUENAT**

**P3**: like I said the supervisory/mentorship has been very encouraging. We have those from IHVN that come to know how far we are doing about the testing, to know if we are having presumptive cases, to know if there is an update on available output that are…… there is platform for that where you update your work per day is and even weekly where we send our report to the platform and from there if there is need for them to add somethings based on outcome of our work per week, they do that. So the work has been supervised effectively and monitored well by the provider. Thank you.

**Question 9. Since Truenat was introduced for TB diagnosis, do you think there have been any added benefit to health care workers or patients. Yes or No?**

**P3**: I think yes. I will say let’s look at aspect of training. There have been prompt training of people using the machine. Since we got Truenat we have had trainings 2 or 3 times which helped us to get more versatile with the machine/work. It has helped us to treat every presumptive case as urgent as we can and see how we need to put in our best to serve the people on time so that their treatment can be also be done on time.

I will also say that in aspect of finance, may be that has not been too superb. Like when we started from TB case findings from the beginning or when we are still doing slide method where you have quarterly review of your slide you know what your incentive will be. After you go for work and kind of treatment you are given. In this aspect, TRUENAT since we started they have been trying but I think we have not had it well as it used to be. I think they can still do better because one thing about TB stuff face in as much as we we want to put in our best in finding case and also see our output is on time. They should be kind of good motivation before implementation of these TRUENAT machine and slide when we started. If we go by records you will discover that the number of lab that does TB training are minute because of your exposure to that sample because many people are actually running away from doing it but when this new innovation that came up with those that I started with myself it was encouraging, you would surely want to work because you know in the next quarter there is something you are going to benefit. It gingered us so when Truenat came in also when training was done, they also promised too that we going to laugh which when they started doing what they said will make us to laugh, we were not too happy about it but late there is a kind of readjustment which we are benefiting a bit from our own facility. The enjoyment is from facility not from Truenat provider so we felt ok. We see it as more of humanitarian service we are doing that is what keep us going on the work. That what I have to say about it.

INTERVIEW: but any other benefit to patients or health workers apart from the training that you mentioned.

P3: to patient I think it has helped them because every of what patients are receiving are free. There is no excuse for any patient to say I don’t want to take this treatment because testing is free and also the cost of treatment. I will say patients are benefitting mostly because if you look back at death rate when it comes to issue of TB since inception of the programme, I think in area where we know it is effective it has reduced death rate significantly so patient will come even when you get a patient that is positive, you still do …. Contact tracing around and every of those things are free to them. So it make them to want to take the treatment because they know that there is no cost attached to it. That is the aspect I see that patients are benefitting from it it is kudos to those that are supporting these programme because when there is no money, there is no treatment because nobody will have a facility and you say you want to run it on charity. So they have benefitted so much. To health provider, I think to us they just make us because there is every in place for you to work with provide your service. You are not slacking in discharging your responsibilities to them because you have everything in stock there is no excuse of you not giving proper and accurate treatment to your patient. You discover that it make you to add a close rapport to your patient it will help in your treatment to them. When you get closer to them it afford the opportunity to encourage the take their medications will. Health officers too has been able to gain more friendship with those people and the likes. The only thing I just don’t know is if there is anything health provider get to give them to get yourself protected against this because there are health providers in the cost of attending to their patients get infected and come down with TB. Such person if there is no adequate care from the provider of these provider of this programme to them they feel that when am not infected I have to relax in my own service to these people so that I don’t contact the thing again that’s the thing I see about it.

P4: yes sir, there is a benefit. The health care workers will get training, seminar and any other kind of things. For the patients, the test and the treatment is free. Most of them can’t afford to pay for the test and even treatment. This is the benefit to the patient.

QUESTION 10: **What are your thoughts about the reliability of TRUENAT test result compared to other diagnostic test?**

P4: It is reliable when compared to the TB Lamp, he only lab platform you can compare Truenat is GeneXpert. You can’t compare Truenat with even microscopic TB lab, TRUENAT is much reliable than those two sir.

TRUNAT is reliable because the result is automated and there is a chip you put inside the machine and it takes a long time to detect if the patient is negative or positive. It takes 30 to 40 minutes. This is what makes the Truenat machine test result more reliable. It is reliable because the machine and test require a series of reagents and procedures that you are going to apply for you to get your result. You have to follow a step-by-step procedure for to get your result and this accounts for reliability.

. QUESTION11: **Based on your interaction with other Health Care Workers (Clinical DOT provider) who provide TB care. How confident are they with result generated by TRUENAT test**

P4: Yes, they are confident because result is coming out from the lab and the person who carried the test is a Lab. Practitioner who is trained and you know that this is a device that runs the test confidently that this result is reliable and they can place patient on treatment based on that result.

**QUESTION 10:** **What are your thought about the reliability of TRUENAT test result compared to other diagnostic test?**

P3: Anytime TRUENAT has helped you to identify a patient as positive and you are doing your follow-up test using sputum smear for AFB, you have to pay attention to your slide critically because you already have it at the back of your mind that this patient am dealing with is a positive case identified by Truenat and need to be monitored very well in such a manner that the patient will have a good treatment package and by the end of six months the patients would have been cured. So I see Truenat to be of higher advantage than AFB. I think the result is accurate.

Sometimes if it is well stained you are not sure of getting accurate result of the patient because of either over staining, may be you over colourized and over washed and the slide has to wash off because you don’t want to go over the whole process again, what remains on your slide is what you will be viewing under your microscope. If you don’t have well defined slide and a well stained slide, there is tendency for you not be able to pick the bacilli the way it appears. There is tendency for you not to be able to score the patient as high as or as low as it appeared on the patients. It is good to help eliminate human error that could have come up in the aspect of you staining or not staining area of that is good for you to smear. You are not sure you have pick the right thing and from there you have missed a case in that patient. In these because the number of sputum you are pipetting into the pre-treatment kit and the likes has been able to help you to have a large volume to work with when you go into large extraction of the gene and to getting into PCR machine which has to do a thorough work on the sputum. It is then that you are sure of getting something more reliable and something more accurate than the way of manual that you are doing but that does not mean that these overrules the manual method because when it comes to the issue of follow-up it is the same manual method which is AFB that we are going to get back to. But TRUENAT has helped you to identify a patient as positive so even when you are doing your follow-up using sputum smear for AFB, you pay attention to your slide critically because you already have it at the back of your mind that this patient am dealing with is a positive case and need to be monitored very well in such a manner that the patient will have a good treatment package and by the end of six months the patients would have been ok. So I see Truenat to be of higher advantage than AFB. I think the result is accurate. That is what I see about Truenat over others I have used.

QUESTION11: **Based on your interaction other Health Care Workers (clinical DOT provider) who provide TB care. How confident are they with result generated by TRUENAT test**

P3: e don’t have really much problem with the doctors that are treating these patients because they believe so much that if patient has given a good sample and they have taken it to the Laboratory, They believe there are competent hands to handle it and being that clinically they also have assessed the patient. So they have something in mind that they are looking upto that this patient with what I have jotted down from my findings, this is likely what is the problem with this patient? When you send your sample to the Lab you are expecting something to support what you have said or something.

Most times we don’t have problem with the medical doctors but where I see that we have most of the problem are people called riders or network officers that are not enlightened about the Truenat machine. Many of them still believe that GeneXpert is more accurate than Truenat machine and because of that most of them even when they have gotten their samples and they are supposed to drop them at designated laboratories where there are Truenat machines to run the samples but they prefer to take it to as far as where Gene Xpert machine is so that they can be able to get positive cases and from my own finding, they spread news around that Truenat is not as accurate as GeneXpert. Most of them, riders, network officers, sprainers are not bringing positive samples for them. All these boils down to the facts that some of them feel that if I have a target to meet for a particular month like a target of ten positive cases, if am able to get the ten, then my salary is going to be intact. So if I did not meet up my salary is going to be reduced because of that most of them feel let me not bother going to Truenat because Truenat is not giving them positive result forgetting the fact that it is what your patient is that the machine will bring out. Forgetting that there are sometimes you can have error due to interference from one sample to the other like I will say recently we had a case of patient with positive case from GeneXpert although low and patient was put on treatment in my facility the second day, that result came out. This patient on her own went to another facility where there is Truenat machine and re-run the test again and it came out to be negative and the patient came back was babbling, quarrelling that we gave her positive whereas it was that at that time we don’t have kit for us to work with. So we collected the sample and send through a rider to where it was run on GeneXpert machine when the kit came in at the beginning of this month. The patient came back with the result she got that she was negative so we have to take back the sample of the patient again and re-ran on Truenat and the result again showed to be negative. So they have forgotten that there are sometimes you can have error may be because of volumes of samples you are pumping into a particular location because you want this rendering the other machine to be insensitive in order to give them enough of what their wanted. Because of this fear of my salary is going to be cut. So from the little time I have been with Truenat I have not gotten a positive case, so rather than me taking my sample to Truenat centre, I rather take my sample to GeneXpert centre. So we have issue on that. Sometimes we don’t have samples to work on or have very little but in some centres samples are packed full simply because one give more positive results than the other thinking that the way GeneXpert work is different from the way Truenat work. That Truenat is not as sensitive as Genexpert. So only where I think we have issue is among these riders or these their head but with doctors that give treatment we have not had issues with them. the doctors, nurses, DOT focal persons do not have issues with the reliability of Truenat results.

**Question:** **Can you think of anything that has been or could be a barrier to effective use of Truenat test for TB diagnosis in your facility**

**P4**: No sir, there is nothing acting as a barrier to use of Truenat in my facility. Everything is working perfectly well sir,

**P3:** The only barrier that I can see, we can actually have is if there is no available kit to work with. However, there have not been any moment we don’t have kit to work with except recent time when the kit expired, that we had to put on hold.

Outside that there has never been a time we ran out of kit to work with. I think the only thing is that if those ‘screeners’ too can also help to always give orientation to patient on how to go about the production of sputum. Some of the screeners thinks if they talk to the patient they will contact the TB. So because of these when a sample is brought they don’t inspect to know if there is quality sample in the cup. There are sometimes you come to work on bench, you will discover the sample in the cup is insufficient or there is nothing in the cup. But if this has been looked into at the point the patient produced the specimen, it will help us better when we work, there will be enough sample to work with for a patient. Even a minimum of 1ml and maximum of 3ml, so that if there is any problem or spillage in the process of running the test, you will still have confidence of going back to collect from the remaining samples. So we need to re-orientate the people in charge of screening; the health providers and also encourage them that by telling the patient to produce enough quantity that will help to get quality and good result for the patient. The screeners and riders should be trained on effective sputum size production because sometimes there could be spillage of samples thereby reducing the quantity because of the way the sample was handled.

The only barrier I see in the use of Truenat for TB diagnosis in my area is irregular power supply. Most times there is inadequate provision of consumables and even data to send reports.

**Report from one of the Participants**

In the Lab sincerely we do not have any challenge in receiving samples or with other colleagues we are working with, reason being that there was a sensitization programme that was carried out during the installation of our Truenat machine, the health workers in the neighbouring health facilities were invited to our facility. We introduced the machine to them and told them how accurate the machine has been .

There was a case of a lady that has all the features of TB patients, emaciated, coughing for long and all the rest of them, when we did it, it was negative. They went to GeneXpert machine it was negative also. So there was confirmation that the machine is actually working. The only agitation is when we keep getting negative result but at a point we start getting positve, everybody was like it is true. If there is positive samples you get positive but if you bring sample that is not positive, you still get the correct result. We don’t have any problem with the doctors or DOT personnel in doubting our result. They all have the understanding because of that sensitization and with the one I told you that happened readily. We have to do sample twice. She went to GeneXpert it was confirmed. There has been smooth running of the machine. Though initially from June to September last year we had heavy supply of samples. We ran above 900 samples in a quarter. So at a point our True Prep machine broken down and that was when we stopped work. It has to be replaced from Abuja before we resumed work. The only time we were out of work, was when our reagents expired, we were asked to cease work until a new supply was made and we have had a new supply apart from…

13. **Can you think of anything that would facilitate or support the effective use of Truenat Tests for TB diagnosis in this facility?**

**Answer.**

**P7:** Provision of solar power to the Truenat machine and regular supply of consumables.

Outreaches will help. The TBL used to go to other places to make samples available to us. We even receive samples from the prison. Then there should be community sensitization besides the health worker sensitization it will also help the use of the Truenat machine.

4. The help of healthcare workers that know much above TB could assist the patient to come directly to the lab for TB tests that will help

5. Increasing the awareness of TB among the people especially in the rural areas will be good. The healthcare workers have a role in play. They should make it known to the people that the TB test using Truenat is free and that treatment for TB is free also.

Question stool sample

3. Yes. I am aware of the use of stool in diagnosing TB in children. My facility has been doing that getting the samples from the Riders.

The only constraint, when you have babies to collect stool samples from. I have asked how long the stool could stay in the fridge before being used. We were advised to place ice packs and sent to lab immediately. I am afraid that because of the pick-up time the stool may not be of use anymore. If we have children to be tested for TB we will treat them as urgent bearing in mind there is a time lapse. We will be glad to do Truenat TB testing for children using stool samples. It will be additional knowledge to us on how it will be conducted and also we will be able to help our children as far as TB is concerned

4. I am not aware but if it is implemented, I will be very willing to use it.

6. I am aware. I will be willing

We should know the facilities that are attached to us for sample provision. This will help us so if we are not getting the samples we will know who to blame. This is because we have people who believe some platforms are superior to the other. So some people in the quest to have more positive cases took their samples to far away facilities abandoning the Truenat sites. S we should know the facilities attached to our center who should be servicing the machine so that our Truenat machine may not be underutilized. This is a joint work, we have the machine but we should be able to receive samples from the neigbouring facilities.

Modification of the machine especially the True Prep since it analyses one sample at a time. If it could be able to analyze two at a time like we have for TrueLab that will be good as that will minimize the time spent working on the machine.

**Title of Interview/discussion: FOCUSED GROUP DISCUSSION (FGD) 2**

| **Interviewer/facilitator/questions** | **Respondent/responses** |
| --- | --- |
| **I think we just have to start** | **All- Okay.** |
| What we should do now is that; you are number 1; is number 2; is number 3; is number 4; I think we can start now. | All- Silent |
| So, we welcome all to this, just for introduction; this is Focused Group Discussion, among Clinicians working around Truenat sites, so we have about 7 questions, so we make do with it. I will see what I can do to contact those who wanted to join but they couldn’t. so I still make use of these numbers; So, I will start with the first question for number 1. | All- Okay |
| **So number 1, can you briefly tell us what you know about the Truenat MTB RIF diagnostic test?** | No.1- Good evening everybody.  All- Good evening.  No.1- Can you hear me?  All- Yes, we can hear you.  No.1- Okay good.  The Truenat machine was installed in my facility for testing, sample, sputum to be precise for TB and the result comes out and is accurate.  Truenat machine is actually meant for use in the low resource setting like ours in Nigeria and it is meant to be kept in a very cool environment, supported by back-up batteries so that it can work and it can go for about 8 to 12 hours, then there are cartilages in there where the samples will be analyzed of suspected cases of tuberculosis. Diagnosis is prompt and treatment is initiated as early as possible.  So, in a nutshell this is the summary of what Truenat machine does or has been doing in my facility. |
| So just as a follow up question no.1**. where, and where have been the sources of these information you are giving us concerning Truenat? Or where did you get or received these information?** | Well, the donor agencies that actually gave the machine and installed it and even repairs it has been giving the information, the support, the training of staff who are involved in the conducting the sample analysis. So it is actually from the Implementing Partners basically. |
| Okay, so we go to no.2. Can you briefly tell us what you know about Truenat MTB diagnostic test? | No.2- Okay I have to brief about my experience with machine in my facility, all I know is that it is cheaper and the result is more reliable. This is because it is least expensive mode of TB diagnosis ever seen. |
| Okay, can you help us with your sources of information on Truenat? | Well, from the donor agencies; Implementing Partners and USAID |
| Okay, so no.3. Can you tell us briefly what you know about the Truenat MTB RIF diagnostic test? | No.3-Good evening everybody.  All-Good evening sir.  No.3. well as for me Truenat just came recently as a result of the necessity because we have been using GeneXpert before now. It is similar to the GeneeXpert and it is also PCR based molecular test. It has the same level of accuracy like the GeneXpert.  Yes Truenat is made for us where we don’t have light and where the temperature does not affect it as it does to GeneXpert. It has been an alternative source to GeneXpert where the issues we have with the GeneXpert is not apparent with the Truenat and so far, it has shown to give the same value like the GeneXpert. Like in turn- around time it is better and it is also good too. So you can do a lot of testing with it over a period of time like in a day, you will be able to do a lot of testing where there is even no light. So for it very-very comparable to the GeneXpert, so it is as good as GeneXpert. |
| Okay, thank you.  No.4, can you briefly tell us all you know about Truenat MTB RIF diagnostic test? | No.4-Good evening everyone.  All- Good evening.  No.4. Truenat machine is a diagnostic machine that recently came on board and is molecular based testing using the PCR for the identification of the bacteria and it is very effective in the sense that the challenges we are facing most especially in the rural settings with reference to light; so it has back- up batteries and rest of them which makes it more convenient to operate in the rural areas and it has improved the consistency of running the test within few hours and it has improved the reliability of the diagnosis especially in the rural settings. |
| Okay, thank you for your contribution. No.5, can you briefly tell us what you know about Truenat MTB RIF diagnostic test? | No.5- Good morning everyone.  All- Good morning.  No.5- Yes...”long pause” |
| I am asking a question sir that you need to respond to. | No.5- Okay, come again sir. |
| I said can you briefly tell us what you know about Truenat MTB RIF diagnostic test? | No.5- Truenat is a machine that produces good quality results and can also determine RIF Resistance. In a facility like that; before we are using some machines to carry out TB testing but now when the introduction of the Truenat came; we have really given out our samples; we have really understood that Truenat is a machine that produces quality results because we have no doubt in the result of the Truenat because it is producing quality results and it can also determine RIF resistance, that is all I know about Truenat. |
| Okay; so thank you all.  We go for the second question and we still go back to no.1..**As a clinician involved in the diagnosis and management of TB patients, what was your experience with the process of laboratory testing for presumptive TB patients before the Truenat was installed in your facility?** | No.1-The experience has been that of delayed diagnosis because you have to send or transfer sample to another town where the tertiary centre is located.. However, this led to the delayed up-take of anti-TB medicines; however, with the arrival of the Truenat machine in my facility which happens to be close to the community; So we now make prompt diagnosis even in the OPD, we have been able to do well in taking care of the presumptive TB cases and we have seen a lot of turn-out; a lot of positive cases because we have this machine close-by in the facility. So it has really helped us in making prompt diagnosis and initiating treatment as promptly as possible. |
| No.2. the experience in your facility before the Truenat was installed sir? | No.2- Okay, before Truenat came our diagnosis was based on testing using GeneXpert machine and we just have one GeneXpert machine in the whole city. In-fact, it was located in the tertiary institution and facilities from and around this city send all their samples to the tertiary institution. You can imagine the samples the GeneXpert center was handling and the time it will take the result to return to us. This was a major challenge. So we now have a new experience with the coming of Truenat that is very favourable, we now conduct the tests in our health facility and the result comes out almost immediately. |
| Okay; no.3. Any response to the same question? | No.3.-Well, before Truenat we had a number of issues with GeneXpert; the use of light; the issue of air-conditioning; the issue of errors; the issue of backlogs, so things were very-very complex during that period but with Truenat; the urn-around-time reduced and we now receive results of TB tests faster. You know before now it took a week, sometimes up to two weeks before the result comes out but now, results are coming faster and there is less error. Please, I am not saying there are errors associated with GeneXpert test but because conditions for the tests weren’t suitable for the machine to run caused the errors. This is not the same experience we have with Truenat. You can take Truenat to the field unlike GeneXpert where you have issues of temperature or things like that. Truenat has improved our out-put and our out-come.  Thank you. |
| Okay, so no.4. | No.4- Before the installation of Truenat machine in my facility; the experience was that we have to send samples to tertiary institutions where they have GeneXpert machine; so there was delay in turn- around time; delay in the diagnosis; delay in the commencement of treatment but now these challenges are no longer there because of the introduction of Truenat. With Truenat everything is prompt hence there is no delay in commencing treatment so Truenat made everything easier. |
| No.5. Un-mate yourself and then talk? | No.5- Okay no.5 on the line “Long pause” the question please! |
| Yes, we are asking what was the situation in your health facility before the installation of the Truenat?  What were your experiences in the management of TB patients? | No.5.- Yea, before the installation of the Truenat machine; Truenat is a machine that produces accurate results. Before the installation of Truenat; we were having challenges even some machines; we were doubting their results; including sputum AFB results. This was because whenever the tests were carried out you see all the results come out positive even when the person is not showing the clinical manifestations of TB. So, we were having these challenges then. However, Truenat changed all that. That is why the installation of Truenat in our facility is very-very important to us, it helps us to have accurate results even to detect RIF resistance. |
| Okay, thank you.  No.1. I will come in with the two sub- sections of no.1 question.  No.1 **Did the Truenat increase TB case finding in your facility? and then 2, you talked about situation before Truenat came; what was the difference Truenat made?** | No.1- Sorry could you repeat the question again? |
| Okay; I am now asking,; what was your experience before the installation of Truenat machine in your facility?  Did it increase TB case findings? | No.1- Thank you. It helped; the coming of Truenat increased the incidence of TB case finding in my facility because there are some cases which would have been lost in the OPD, if not that we have that equipment in the facility. So we were able to pick them quickly at the OPD and put them to test on and to our greatest surprise; we were seeing many TB cases unlike what it was before then when we needed to narrow down the cases for GeneXpert test in the neighbourhood facilities. Now with high index of suspicion; we are able to pick some presumptive cases have them tested and within few hours you will be able to get the results. Truenat has helped us pick up many cases of tuberculosis that we could have missed and we edstart them on treatment immediately. Thank you. |
| Okay, thank you. So we are taking these two questions for everybody. No.2. Just, tell us the experience after Truenat was installed?  And again; did Truenat increase TB cases in your facility after its installation? | No.2- With the installation of Truenat in my facility, TB case finding has increased in my facility tremendously. This is. because we also get samples from the community outside my facility apart from those who come to the OPD, because people have realized that Truenat is a very fast and reliable method of diagnosing TB.  Facilities now send their samples for us to run the test; it has actually improved and increased case findings and generally it has provided the impression to our patients that they can get their results as quickly as possible... |
| Hello! We are not hearing you sir. Okay, let me go to no.3. | No.3. I will say that it increased TB case finding because we have the opportunity of testing more people and these were samples that otherwise would have piled up in GeneXpert sites waiting to be tested. Truenat remarkably reduced the delays associating with testing and obtaining test results making it possible to start treatment for patients on time. |
| Good, thank you.  No.4. Your experience after the installation of Truenat machine in your facility? Did it increase TB case findings? | No.4- After the installation of Truenat machine in my facility, the number of presumptive TB cases identified at the OPD unit increased because they are no longer sent to other health facilities for test. The Presumptive TB cases are tested immediately at the facility. So sample testing has increased and also TB case finding. Most importantly, tests results are received almost immediately. |
| No.5- Un-mute yourself and talk.  The experience in your facility after the installation of the Truenat machine; did it increase TB case finding? | No.5- With the coming of Truenat machine to my facility, more people are being tested for TB, the results come out on time and we also start treatment on time. Many people are now coming to the facility to be tested since they know that the test is free. Again, there is a certain health facility that tested 25 samples as positive for TB but when the same 25 samples were tested using Truenat, only five of them were positive. So you see that Truenat is very important, it is portable and also gives accurate results so patients are managed properly. Thank you, Sir. |
| Thank you.  So we go to the third question; I will still continue with the same order: C**an you share your perspective on how the Truenat machine has affected the diagnostic journey on the experience of patients undergoing TB testing in your facility**?  No.1 | No.1- Okay sir, Truenat has reduced the time for testing for TB and the waiting time for patients has reduced now when compared with the previous time they had to wait for days or even a week before getting the test results. Patients can come in the morning for test and will be able to get their results same day and placed on treatment immediately. So, Truenat has afforded us that opportunity of prompt diagnosis and prompt treatment and then waiting time for patient has reduced considerably and this is an added advantage. With more testing, we are able to detect more cases and start them on treatment hence curbing the transmission of tuberculosis in the community. Thank you. |
| Okay, no.2; can you share your perspectives on how the Truenat machine has affected the diagnostic journey or experience of patients undergoing TB testing diagnosis in your facility? | No.2- Truenat machine has really improved TB testing and case finding in the facility. Once samples are received, the result come out almost immediately and from there the patient will be placed on treatment. The test results coming out fast is an improvement on the side of the health facility and good experiences on the side of the patients.  Thank you. |
| Thank you. No.3 | No.3- For me my perspective is that Truenat has come to enhance our services as we are able to give our patients prompt response; delays are far reduced; testing is done as quickly as possible and results are received at once so patients access their treatment faster. You know one thing with people here is that once treatment is delayed, they search for alternatives. So with Truenat, we attend to our patients promptly and that is good. |
| Thank you sir.  No.5 Share your perspective on how Truenat machine has affected the diagnostic experience of the patients undergoing TB testing/ diagnosis in your facility? | “Muted” |
| We may have just to move on as a large number of us are having good network services.  No.1, I will get back to you maybe I will just maintain this order.  **How confident are you in using Truenat results to make clinical decision for a TB patient? As a follow up question; What are your thoughts about the reliability of the Truenat test results compared to other diagnostic test?** | No.1- Thank you; the reliability of the results from the Truenat machine is very good and specific and if compared to GeneXpert is something at par with the level of result it is giving; however, I think it is better than what we used to have on time saving when compared with “AFB”. The result is specific and the specificities of the result from Truenat machine is what one can rely on; so it is reliable.  Thank you. |
| So, you are confident using the results from Truenat machine to make clinical decisions for a patient? | No.1- Yes, very confident in using it. |
| Okay; thank you.  No.2 | No.2- I am very confident with the result from Truenat to diagnose patients for TB and that confidence sets from the fact that it is not just looking for the anti-bodies but it operates at a higher level with high level of reliability. So it is at the same level with the GeneXpert. So, the test results are very reliable and we make good use of the results in making clinical decisions for patients. |
| Okay, thank you sir.  No.3. | No.3- The fact is that I am confident using Truenat results since its MTB/RIF test is gene-based and its sensitivity is as accurate as GeneXpert. I am very confident using it. |
| No.5. | **No.5-** Yes sir, I am confident in using results from Truenat machine because it is a machine that is very easy to operate and it is producing better results than other testing platforms. I am not saying that it is better than the GeneXpert but this Truenat machine is producing very good results; it is very easy to prepare a sample; very easy to carry out the test and in summary I have confidence in using the results.  Thank you. |
| Thank you. No.1, I am coming back to you; I am maintaining that other.  **Since the Truenat machine was installed in your facility, can you tell us the challenges you were aware of with the maintenance of the machine or that process that significantly affected the services to the patients been evaluated for TB?** | No.1- Thank you.  The challenge we have with Truenat in our facility is that of power. We do not have public power supply and we do not have a generator. So there is the problem of charging the battery attached to te machine. Again the consumables do not come in as at when due so sometimes we run short of consumables. |
| Okay, thank you.  No.2 this question is for you sir.  Since the Truenat machine was installed in your facility, what are those challenges that you are aware of concerning the machine that significantly affected TB diagnostic service delivery to patients? | No.2- Since after a while the challenges after the installation were overcome; everything was running smoothly until couple of weeks ago when we started getting some error results or invalid results and we placed calls across to the relevant stakeholders and then we found out that it is related to our supply system. So that time all the samples we submitted or supplied to work on; we didn’t get any result at all. They were just error messages or invalid results. So they have to come back, change the box and after that the results started coming out very well. So these are the challenges that have come to my own notice. |
| Okay; thank you sir for that submission.  No.3 | No.3- I can’t imagine any issue like that rather I would think of the human factor there; because they have been increased loads; so it is more of the human not the machine itself and then the issues with consumables; yes the human factors, there are some individuals whom I don’t know what their problem is; there is this staff that is always having error using the machine, I think it is a human factor not the machine and then the consumables that maybe sometimes are not available as at when required because there is an increase load of work now due to the use of the Truenat machine. So out of these, the machine itself is not giving us any problem as such; we have our personnel that we have been having issues with the machine and we are working on that. |
| Thank you sir.  No.5 | No.5- Yes sir; for me challenges from Truenat machine since it was installed in my facility. I didn’t see any challenge apart from the power supply because where I am we have no electricity and there is no generator and that’s the big challenge; Another thing is consumables; things we would want to use like hand gloves, facemask and the rest like detergent, bleach for disinfection but apart from these we have no other challenge because the machine is functional unless it has problem or fault but for now the machine is very-very functional. |
| Thank you.  **No.1. What do you think could be done to improve service delivery from the Truenat machine in your facility?** | No.1- Thank you.  For the Truenat machine I think we have had discussions about the machine with the donor agency on the possibility of installing the solar panel that will be supplying power as the main source of power to Truenat machine such that the power supply will be suitable and constant from the solar panel apart from the national grid as an alternative source. So this is the issue that we have been able to discuss with them and they will respond when then they feel convenient to do so.  Thank you. |
| Thank you.  No.2, what do think could be done to improve service delivery using Truenat machine in your facility? | No.2- My contribution was actually based on what the last respondent has said. There is need for a solar panel attached to the Truenat machine for the purpose of charging the machine. For a disease like TB, collection of samples and preservation of such samples cannot be done in a very tight room; in a very small room; even the health personnel are not finding it easy. It will be good if the room is expanded or another one built for the health workers that will help in improving service delivery. Then, there are just few people in my facility as Lab. Scientists who know how to operate the machine and if something happens and most of them are not around the Truenat test pauses for a while. So training courses should be organized where other Lab scientists should be trained to enable the knowledge go round; Training should not be just once. There should be training and retraining that can last up to 5 days for them to get more knowledge on how to operate the machine. I think more people should be trained so that in an event that one person is not around another can operate the machine or as one who works under government, transfer can occur at any time to another facility and the person who is been transferred from the other facility may not have knowledge about the machine and the person who was handling the machine might be taken to where there is no machine. So I am thinking that training should be probably more frequent to ensure that the machine is not closed down one day. . |
| Okay, thank you sir.  No.3 | No.3- From my side, they are always shouting for incentives, yes that is what they need; they have been crying that they are the least supported as in financial support. Secondly, we should employ more staff, Also, since transferring trained personnel in government service from one health facility to another is not helping, we could have support staff from probably the partners coming to support the government workers so that in-case of a transfer, somebody is there to man the machine day and night; then we will be able to run more samples and get more results from the Truenat machine. So incentivising those who are working there and then having more staff to work in this lab is important.  Thank you. |
| Thank you sir.  No.5. Can you hear me;  No.5 can you hear me. | “Muted” |
| Okay, I think that question is actually the last question; then coming back to no.1. Is there any other you thing you can say about Truenat machine that was not included in the interview guide that reviewed so far | No.1- I think there are some gaps but what I really appreciated was that they want to make the diagnosis easier with this portable testing machine. So, I am advocating for the personnel to devote more time to the machine as this will make diagnosis easier in the facility. So outside that all other things have been mentioned like the issue of power supply so that one can have power supply and available consumables. I think that’s all.  Thank you. |
| Thank you.  No.2. Any closing comment; anything you feel we didn’t directly enquired about | No.2- I want to say support with provision of the machine should be made more available for us in the country; I know it is not easy but our supporting partners can help to make the machine more available because it is more reliable and cheaper alternative to GeneXpert. It should be made more available in our communities to actually improve diagnosis of TB cases. So all facilities should actually go for more machines for effective TB diagnosis in our communities.  Thank you. |
| Thank you.  No.3 | No.3- I didn’t have really much to add but if the machine can be made more available in the health facilities. I don’t want to say replacement for GeneXpert” because there are a lot of support for GeneXpert that Truenat machines don’t really need; so if we make Truenat more available in our health facilities to give more support to the laboratory staff and then these consumables surrounding the use of the machine should be always made available because it will go a long way to sustain the use of these machines in making diagnosis for TB patients and RIF resistance in our communities.  Thank you. |
| Thank you very much.  So, we have come to the end of this discussion. I must thank the participants for their resilience and the spirit that was on all through the end of this discussion. | All- thank you sir. |
| Good night. | All- Good night sir. |
|  |  |

**TITLE OF INTERVIEW/DISCUSSION: FGD 3**

| **INTERVIEWER** | **RESPONDENT** |
| --- | --- |
| Just say accept and then we start. God afternoon all. | (All): Good afternoon sir |
| Thank you and you are all welcomed. We have talked so much about this so I will not enter into the nitty-gritties. So all the rules we have made and all the information we have received before this remain valid. So I will start up with the question.  We understand that this is a Truenat operational research focus group discussion among State Quality Assurance officers. So you are all welcomed and thank you for participating. Most importantly also, thank you for coming on time. I appreciate that.  The last person that came in now, you are number 8 so we are starting. | I am still the one, I am also using my laptop |
| Okay, so we are 7 in number. I am starting. | All- Silent. |
|  |  |
| **What has been your experience so far with Truenat test for TB diagnosis in the state- in your state? Can I start with number 1? Just tell me what your experiences have been.** | No1-Good afternoon sir.  In my state, we have one Truenat machine. My experiences with regards to Truenat is very awesome. Truenat machine is one of the modular test used to diagnose TB and Truenat is a rugged machine and it is also very simple to manipulate. It also helps the laboratory personnel to be able to run the samples with ease. Truenat is rugged; rugged in the sense that it does not need air conditioner. If you want to compare it with GeneXpert machine, it does not need air conditioner. A little light without AC, it works. Truenat machine can differentiate between MTB detected that is MTB and RIF resistance detected that is, if there is in the sample. So Truenat is very important and it helps us to increase cases in TB findings in my state. So Truenat is a good molecular machine that helps us to increase our cases in the state. And it helps the TB staff to work with ease. Once you present your sample, you can go and do other things. So you will have time to do other things if you are using Truenat. I think that is my experiences with Truenat machine. |
| Okay, thank you. Thank you sir. Number 2. What has been your experience with Truenat machine | No.2- Good afternoon all. The experience I have with Truenat machine; I am speaking from my state; is that it is a good machine. We have 3 of Truenat machines in Oyo state presently and all the three sites are doing well with the machines. The only challenge we have with the machine is that we have to detect MTB first after which, we have to start re-running the test for RIF resistance thereby wherever we have positive samples, we have to do it four times before we can determine whether it is RIF resistance or not. Apart from that, the machine is good. The turnaround time is also good compared with other molecular tests. It is only taken less than an hour to detect MTB whether it is positive or negative. Then also; the atmospheric condition under which it works is very friendly. It doesn’t need much electricity or much power to work or structures like air conditioner to work. It requires minimal infrastructure in a room where there is a bench and minimal electricity coming into the room, you are good to go. That is the best I can say. Thank you. |
| Okay. Number 3 | No.3- Long pause |
| No 3 are you there? | No.3- Good afternoon everybody. I want to say that Truenat machine is a very rugged machine. Rugged in the sense that it facilitates the diagnosis of tuberculosis. It requires minimal infrastructure. It requires minimal education of the personnel to operate it. It is very simple, and if MTB is detected, you go ahead to carry out your RIF resistance. In other words, if you diagnose TB successfully, initiation of treatment is straightforward and within a very short period of time. If a RIF resistance is dictated, (because if you get MTB detected), you will confirm the real status. If it comes out to be RIF resistant, you will know the proper treatment to initiate. So Truenat machine is very rugged. Just like the other two speakers have said, it does not require any air conditioner and that is why it is fantastic in the rural or remote areas. We have three (3) in my state, in 3 LGAs. It is easily carried about so it facilitates community testing. I just love the machine. Thank you. |
| Thank you. Number 4. If you are not in a noisy place, please can you mute yourself? We have much interference. We are recording. | No.4- Long pause |
| Number 5 | No.5 - Good evening all |
| Somebody is having poor network issues. Please the person that is speaking should stop, we are not getting you; the network is poor. | No.5- I said we have two (2) Truenat machine |
| Interviewer cuts in: The person that just came in should take Number 9. The network is bad. Somebody just joined us now, please pick number 9. Okay Number 6 | No.6- Okay number 6, I am speaking from my state. Can you hear me? |
| Yes, I can hear you. Thank you | No.6- Okay. We have two (2) machines in my state. The Truenat machine is the rugged one like everybody have said and you can use it in a place where you have minimal infrastructure. But like the second or third speaker said; there are two limitations in the sense that: firstly, the diagnosis of TB being detecting MTB before going ahead to detect RIF resistance, the MTB plus is when you have finally tested and you have gotten the MTB before you go ahead to start processing for the RIF status. It takes some time.  Then secondly, if there was a way whereby you will just go straight in detecting all like the GeneXpert system does; I think it would have been a better one. Thirdly the processes of extraction before going through labs and through prep and all that. It delays, its time consuming and all that. So most of the time, the people in the site, you have to be on ground unlike the GeneXpert whereby once you load the system, you know you are through with that and you go ahead to do other things. So if there was a way whereby Truenat could be two-way: you do this one for a short period of time, after sometime, then you come again and do the other process. In all, when we put those other things aside, I think going ahead to the rural areas and all that when we think of the infrastructure and the rural area(this is where we have facilities without personnel because in our time now where we have maybe one health worker minding this and also minding that?. Because personnel is a problem in most of the facilities. If it is a place where we have more one health worker minding this and minding that, it is a challenge to some extent. So those are the few challenges we see on sites concerning Truenat. |
| Number 7 are you there? Okay number 8 | I am number 7, The experience with Truenat in my state: we have 3 Truenat machines. It has not been bad, t has been a good one. With Truenat machine, there is an increase in our case detection but there are some limitations when we compare it with GeneXpert. Comparing it with GeneXpert, one of the limitations is that it cannot detect RIF resistance and MTB together at the same time. After detecting MTB, you will still have to go back again and start processing for RIF resistance. It is a great limitation. Then the hassle is much when it is being compared with GeneXpert. With GeneXpert you just need to mix the sample, go there again mix the module time with different intervals and load the sample and wait for your result; but for Truenat, you have to be around all through the time of the processing. And most of the laboratory we have in Osun state is a general lab, whereby you have to also process other samples other than TB samples(though I said the experience with Truenat has been good because the cost of maintenance is low compared with that of GeneXpert). Then the TAT is not bad, it’s not too long when we want to compare it with microscopy. Truenat machine is rugged and it is friendly in terms of the user. Then the atmospheric condition- requirement is very minimal. It does not need AC before it can perform; it does not need too many inverters only needs full batteries (solar battery or back up like generator to charge the batteries) and continue with the work. So, the problem with maintenance is minimal when it comes to Truenat machine. The TAT is still okay though we have 2-module Truenat machine in Osun state and the number of samples it can process is not as much as GeneXpert per day. The work load is okay and it is faster and easier when we compare it with microscopy. It can detect MTB and RIF resistance. So the major challenge is if there is a way it can be modified to detect MTB and RIF resistance at the same time without one having to go back again and start the processing, I think it will be nice. Thank you very much. |
| Thank you madam. Number 8 | No.8- Good morning everyone, I am from Lagos. I am sorry I don’t think there is any different thing I’m going to say that will be different from what all my colleagues have said from their respective states so that we don’t start wasting time. However, we have 5 Truenat facilities in Lagos, one is privately owned. The others are owned by the programme. I think just to summarize everything, we are looking forward to an operational plan whereby Truenat – the turnaround time will be reduced and it will be able to detect immediately; not to go back just like my colleagues have explained. Thank you. |
|  | In my state we have three Truenat machines and all are in the primary health centers. The machine is rugged, it can detect mycobacterium tuberculosis and also determine rifampicin resistance and that means complete diagnosis of TB be it Drug sensitive or drug-resistant tuberculosis. The limitation of the machine are as follows:  a). The two modules are the ones installed in Nigeria at present, this unlike the GeneXpert which has four modules, reduces the number of samples that could be tested in a day.  b). Truenat can only analyse sputum samples hence stool and other extra-pulmonary samples cannot be used to diagnose TB.  c). Only serial testing is supported, ie you first detect TB then carry out another test (MTB RIF Disease) to determine the Rifampicin resistance status of the TB unlike GeneXpert that can detect and determine RIF Resistance simultaneously. Apart from these limitations, the machine is okay. |
|  |  |
| Okay. I have been warned that this meeting will stop in 10 minutes time so remember that we will come back immediately if it stops. Okay number 9, I will start the new question with you. How has your state TB testing, case finding and service delivery be affected by the introduction of the new Truenat machines? Number 9? | No. 9- Can you hear me? |
| Yes sir. | No.9-If you can hear me good day to all of us |
| Thank you. | No.9- So, I want to thank you especially for having this meeting. I am……. So and exactly what the last speaker said; it is just the same thing. We have just one Truenat machine in my state and it is situated in a hard-to-reach area. |
|  |  |
| **Sir you will go to the next stage. I said how has the TB case testing, case finding and service delivery been affected by the introduction of a new Truenat machine in your state?** | No.9- Okay, it is positive. It helps tremendously |
| Okay, so has it affected TB case findings? | No.9- Yes, positively |
| Any evidence? | No.9- From the report I sent to National, I cannot recite it off hand. |
| Okay, thank you. So how has your work been affected by the introduction of the new Truenat machine? I am still continuing with you, number 9, how has your work been affected by the introduction of the Truenat machine in terms of TB test and TB diagnosis? | No.9- It helps us (Interviewer cuts in) |
| Has it affected your work load? | No.9- Yes, it affected and it has increased our work load |
| Are you busier now than before Truenat came? | No.9-Yes, it has lessened our burden |
| But are you busier now than before? | No.9- We are busier now. |
| Okay | No.9- We, the health workers are busier now with the introduction of Truenat and presently the health workers we have are few in number. In the health facility where we have Truenat, we have a lot of samples, the Lab staff works all through the night this has increased our case finding positively. |
| Okay. Thank you. I will be using numbers now so that we may be jumping some things. it seems like in the next ten minutes, we may be cut off to rejoin. | All – Silent |
|  | Truenat has helped to reduced TAT, result is received very fast, It is accessible at the PHC settings, Patients now have direct access to testing, there is increased trend of community notification, and public-private mix as one of the machine is in private facility.  Reduced sample referrals, I need not to ensure coordination of sample movement to GeneXpert site, testing is been decentralized |
| So, number 6, how has your state TB testing, case finding and service delivery been affected with the introduction of the new Truenat machines? Number 6 | No.6- Okay, I will say it has affected it positively in the sense that the Truenat machine; when it came, we put them in places where we didn’t have GeneXpert machine. |
| Okay | And those Truenat machines were sited in LGAs there were no GeneXpert machines – which they had to transport samples from those Local Government to GeneXpert sites. So we know that the Truenat machines are there now: instead of moving samples from there to other Local Government, they are now running the Truenat machine and even receiving samples from GeneXpert sites and other people. I think from the statistics we have, they have been doing tremendously very well. It has been helping us positively. Even so many RIF resistant cases have been detected at the sites. |
| So how about your work load? Has the Truenat machine increased or decreased your work load? | No.6- Like in one of those sites, it has, until their machine broke down towards the end of last quarter and the beginning of this quarter. But for the second site, it has increased it tremendously. |
|  | Our workload would have increased, but in the other way round we experienced equipment down time with other molecular techniques, also lack of reagent for the Truenat machine for almost a quarter. All this have made it difficult to explain how it affected our workload.  I am less busy, because 3 of our the LGAs where the Truenat was deployed were actually without any means of molecular test, Most at times samples were referred to other LGAs, challenge of sample movement in that LGA were drastically reduced |
| Okay, so let me go to number 2. Number 2, it is still the same question but let’s see if we can finish it before we are cut off. So how has your state TB testing, findings and service delivery been affected by the introduction of new Truenat machine? Number 2 sir. | No.2- Good afternoon all once again. Well, the Truenat machine has increased our work load in that, in the state capital, where we have many GeneXpert machines, we now have less load to work on; we now have less samples to work on because the Truenat machines are mopping up the samples that would have been referred to the state capital. The Truenat machines are mopping them up in the other LGAs where they are situated. Like in our state we have three LGAs where they are situated so sample meant for RIF resistance are no longer transported from all those three Local Government; they are done in those three LGAs and they get their results on time within their LGAs within a short time- maximum a day, within twenty-four hours. So, there is no need for transporting samples to the state capital for GeneXpert. |
| Has it increased TB case findings? Still number 2 | No.2- Yes, it has increased TB case findings. In fact, the last report for quarter One, 2023, we had RIF resistance in one of our facilities where we have Truenat machine. It has increased TB case findings. |
| Work load, has it increased it or decreased it? | No.2-It has increased work load in the facility where the Truenat is but it has decreased work load for facilities that are in urban areas especially those with GeneXpert machines. |
| Okay. That is wonderful. I am sure we may be cut off any moment from now. But remember that we will join again please because we have not finished. | All- Silent |
|  |  |
| **Okay I will go to the third question. I will be picking numbers. We want to move faster now. Number 3, how well do you think the Truenat test for TB diagnosis has been successfully integrated into the TB diagnosis network of your state? Number 4** | No.3- You said number 3 |
| Okay, number 3 go on please | No.3-Very well in my state. We have three Truenat machines and they are located in the Primary Health Care centers in the Local Government Areas. Truenat machines since they came have been well integrated into the TB diagnostic network in my state. Because the Truenat machine as I said before is rugged. Samples in the rural areas from ‘hub and spoke’ are moved down directly to where the Truenat machine have been installed for analysis. The analysis is done. So it has now reduced the time of moving samples from rural areas to cities where before now it is not as it used to be. So Truenat has in that regard made testing complete. We don’t need to test and get MTB when tuberculosis has been detected; as soon as tuberculosis has been detected, we begin to move samples for another round of testing to ascertain RIF resistance. No- the Truenat machine is well able to detect RIF resistance. So, if tuberculosis is detected, the real status is determined and the appropriate treatment is initiated within a shortest possible time. Thank you. |
| Thank you. Number 8 | For my state, we had two RIF resistance detected from using the machine and we had 148 cases of MTB using the machine. Though we have some that are also in determining- we had about 26 MTB detected and RIF resistance indeterminate. So, it has been well integrated in my state. |
| Okay. Number 1: how well do you think the Truenat for TB diagnosis has been successfully integrated into the TB diagnosis network of your state? Give us explanation please. | Ok. Truenat machine or Truenat diagnostic network has been successful in my state of operation because we are getting data- a good data from Truenat; though we have some few issues with regards to sample transfer or sample shifting in that area. We have not experiencing samples the way we want but we have integrated the testing into our own network by getting the data that is coming from the Truenat machine to add up to our case finding in the state. So Truenat is adding more case in our cases in my State and we have taken note of the data that we are getting from Truenat. Truenat has already been integrated in our state data, so are working with Truenat very well and it is helping us to increase our cases in my state. Thank you. |
|  | The introduction of Truenat machine for TB testing has really increased TB case finding, increased patient access to these facilities and reduced TAT compared to the time when samples were taken to GeneXpert sites. Thus case finding has improved tremendously. Also, my workload has increased because I have to also oversee Truenat sites in addition to GeneXpert sites. My workload has increased since the Truenat machine has been introduced for TB diagnosis in my center. |
|  | Truenat is well integrated, since the performance of the Truenat MTB, MTB RIF DX show comparable accuracy to the Xpert MTB, MTB RIF. Again, its introduction in the country had the approval of the National TB Control Program and other TB stakeholders in the country. |
| Okay. Number 8, I will end this question with you. I know you are here now. How well do you think the Truenat test for TB diagnosis has been successfully integrated into the TB diagnostic network of your state? Give explanations. | Okay thank you sir. It has been successfully integrated whereby for us in Lagos what we did is that the machines were deployed and installed in facilities that did not have GeneXpert or other molecular diagnostic tools. Those sites were able to help us prioritize testing from the respective LGAs. So all TBLS there are aware of the fact that these tools are in their LGA so for them what they do; because in Lagos, all LGAs has GeneXpert, so what we do is just like a stop-gap measure whereby when samples are excess from a particular section for those that have four modules, we prioritize by sending samples to the Truenat labs and the labs are able to quickly diagnose using their machines and they records. We have a platform where all their weekly data is sent to; then we are able to know how many test we did, how many invalid, how many indeterminate, how many MTB they detected. So, we are able to monitor that in the platform and all along it has contributed and scaled up our in our diagnosis. Like now, I think we have 38 cases detected using Truenat. So, it is a plus if you ask me. |
|  | Truenat diagnostic test has been very integrated successfully into the TB diagnostic network of my state and Nigeria. The machine has been well accepted by healthcare workers. This is because a lot of awareness creation was done before the installation of the Truenat machine. Sensitization and awareness creation were carried in the LGA in preparation for installation and the General healthcare workers have been informed that it could diagnose Tb just like GeneXpert, do it is generally accepted. |
|  |  |
| Thank you for that. What we will now do is I will be picking numbers for a specific question. However, if you know you have a response already prepared for any question that you may not be called to answer, you can send it to me. I will add it up as part of the deliberations from this interview. So I will go to the fourth question.  Number 2 I will start the question with you**: practically from your supervisory and mentorship visit, what has been the reported experience of the lab staff from the use of the Truenat machine for evaluation on presumptive TB samples compared to other TB testing platforms that they have had experience with including microscopy and GeneXpert? Number 2 please.** | Good afternoon sir |
|  | The reported experience has been with limited supply of power to charge the battery, the availability of only two modules for carrying out only two tests per batch so workload increased since these facilities are still carrying out microscopy test for follow up, staff attrition as transfer of staff in LGA is very rampart and they are few.  a). Truenat has two pieces of equipment (Trueprep for DNA extraction) and Truelab (for amplification and detection). You have to first extract the DNA before you start amplifying the extracted DNA to move on unlike GeneXpert that I straightforward and all the procedure from DNA extraction to detection is done by the machine, the staff has to carry out these procedures one after another.  Conducting maintenance is okay, quality of result is very okay since it is a molecular diagnostic test . In terms of the throughput only 24 samples can be analyzed during the working hours while GeneXpert can analyze up to 48 samples during the working hours of a day. In all ramifications, Truenat is easier to use than Truenat. |
| Okay, thank you. Good afternoon, did you get the question? | Yes, I have gotten the question. The experiences from the supervision is that they feel it will be better if we can have a quadro machine instead of the duo that we have in their facilities because of the quantity of samples that they run each day. With the Truenat, they are only able to run about 10 samples maximum in about 12 hours a day. But if you have a quadro that means about 20 samples in a day. That is their experiences. And also, when we compare it to microscopy: when they do microscopy, mostcenters can run up to 40 AFB microscopic samples in a day. But with Truenat we are only able to run up to 10. So, they will prefer a situation where they can have a quarto machine so that they will be able to catch up with other GeneXpert machines that run up to 40 in a day. Thank you. |
|  | In terms of ease- of - use, Truenat is more complex than Xpert as it has multiple hands-on  Most times the operators will say they have carried out Preventive maintenance but no record to show but as a QAO I am trying to ensure that I document all maintenance carried out on the machine  The good news is that the performance of the Truenat MTB, MTB RIF DX show comparable accuracy to the Xpert MTB, MTB RIF it’s on record. The TAT is reduced because results can reach the owner at the same day  However, the Throughput in terms of number of samples is not adequate compared to number of hours the Lab staff put for testing daily. This makes it necessary that the quarto machine should be introduced in the country. |
| Okay, so a follow-up question: in terms of (still number 2) ease of use, conducting maintenance, quality of result, turn-around time how is it in terms of number of tests? | Well, in terms of turn-around time; the turn-around time is very good; quality of result is good too and is reliable. We do not have any cause to doubt any result from the Truenat machine: maybe the patient came out with positive and we are doubting it and we have to re- run it using another diagnostic tool that is obtainable. Whatever the Truenat machine result is, is what we got with other TB diagnostic tools. |
| So, I will need two more people to talk on this question 4 so that we can move to another question. Number 3: practically from your supervisory and mentorship visits, what has been the reported experiences? Number 3 are you with us? | Thank you so much |
| Are you having network issue? | It’s a bit better now, can you hear me? |
| I: Okay, I think we are having a network issue | NUMBER 3: From my state, the lab staff are happy using the Truenat machine as a diagnostic molecular test and the results are reliable. The procedure for maintenance is |
| Okay the network is bad, so number 5 can you take up from there? I think we are having issues with number 3 on that number 4 question. Number 5 do you have the question with you so that you can go ahead and answer question number 4 | Did you say I should answer the same question you asked her? |
| Yes. Practically from your supervisory and mentorship visit, what has been the reported experience from Truenat lab staff on the use of Truenat machines for evaluation of presumptive TB samples compared to other testing network that they have had experience with including microscopy and GX? | For us in my state, the experience has been awesome. The work load is high. There is no much issue with their maintenance only in the last quarter that they ran short of reagents which they have replaced. But the machine we have in the facility has been giving us problems, even now We have gone there together, I and my TBLS, that is the supervisor to see how the situation is. But anytime we go there, we will fix the problem; when we go back, the problem will come up again. But apart from that site, the GeneXpertis excellently doing well. Comparing them with GeneXpert. I can say that the only advantage the GeneXpert has over Truenat is it can detect rib resistance concurrently but nevertheless, the time that Truenat will be used to detect MTB is not much and we don’t have challenge with that. Just as my colleagues have said, the maintenance is not difficult except for the machine engineers are aware that have been malfunctioning. And comparing with TB LAMP, you know TB LAMP does not detect RIF resistance, it only tells you whether there is MTB or not. If there is, you have to send it to the nearest site to ascertain the real status. (ie RIF Resistance)Truenat is very excellent. We don’t have problem with that and we are enjoying the machine very well. |
| Okay, you have already answered the second question at once. Okay number 6. Please can we off our video so that it will not affect our networks. Can we off our video so that it may not affect our networks? | Yes |
| Okay, so practically from your supervisory and mentorship visits, what has been the reported experience of the lab staff on the use of the Truenat machines for evaluation of presumptive samples as compared to other TB testing platforms that they have had experience with including Microscopy and GS? | Okay. For them they said they are okay with it but like I said before; the only complain is if it was more than a two-module where they will be able to conduct more than two test like up to four tests. That is one of the shortcomings that they are complaining about. The other issue is not going with the different processes involved in the procedure at once so the process is not straightforward. If not, in all, they are okay with it. But if there are different ways they can make them as simplified as possible, they would love it more. Then they should try as much as possible to increase to four-module so that at least doing a lot of work like this, they should be able to test as many samples as possible at the same time. Those are some of the complaints they are complaining about if not the maintenance is very okay, just occasional from time to time, they check up on them, they are very okay. So, they are comfortable with that. |
| Permit me to still ask the ‘B’ part of it: in terms of ease of use, conducting maintenance, quality of results, turn-around time; how is it in terms of number of tests | How is what? |
| The ---- | Compare to the number of test, the number of test for the two sites like for my center- they test more because we have two facilities and you know that dedication matters a lot. Some of them when they are on call, they put more efforts as much as possible to do more testing there. Like one of my colleagues rightly said, there is nobody that is actually saddled with the responsibility of doing TB work. As you are there doing this TB work you are at the bench with other works. So, you are doubling as the TB focal person and at the same time doing other works. So, you are not just doing this only. When they are on calls, they will be doing other things but in all, their work load is quite commendable. |
| Okay, thank you. Number 7 I will end this question with you so that we can start the new question with number 8. So practically from your supervisory and mentorship visits(that is number 7), what have been the reported experience of the lab staff on the use of Truenat for the evaluation of presumptive TB samples compared to other TB testing platforms they have had experience with including microscopy and GS? Number 7 please. | In my state, the experience has been good. When compared with microscopy- you know microscopy has a turnaround time when being compared- it is more than that of Truenat but when compared with GeneXpert, the experience is that GeneXpertis lower than that of Truenat. GeneXpert is less cumbersome but these Truenat machines, in fact one of them is installed in one of our hard-to-reach areas (that hard to reach Local Government Area) and it’s the machine we are using to detect our TB in that Local Government. It has been helpful. The challenges we are having with them is that we experience occasional breakdown of the machine which can take one or two weeks to be repaired or replaced. Then last quarter, there was shortage of reagents on the machine. So the experience has not been bad. I want to encourage that instead of duo machine, they should give us quadro because the number of presumptive samples we are generating in my state is much. So we need quadro and as I have said earlier, if there is a way the machine can be merged whereby it can detect both MTB and RIF resistance at the same time, it will be fine. Then another experience we had in one of the facilities is that the facility doesn’t have constant supply of electricity; so, after charging the battery, you will use it for a day or two days and that will be the end. So, they will be waiting again for electricity supply. If they can have a back-up like solar battery that will be used in charging the battery of the Truenat machine, it will be better and helpful.  Like I said, one of the machines is located in hard to reach area, the machine cannot be used to test extra pulmolnary samples like stool which is very important in diagnosing childhood TB. That means that if they have stool samples in such area, they will have to carry the samples to GeneXpert sites. So, all these should be looked into. Thank you. |
| Okay, thank you. Please if you are not talking can you mute so that we can be able to move on? Number 8 I will start number 5 question with you. For those questions that perhaps you did not respond to, you already have answers to provide the response, you can send it to me via whatsapp and I will include it as part of this discussion. Number 8: I will go to the fifth question so we can move on. **Can you share your experience during supervision on how the Truenat machine informed in the field in combination with the PDF,CSD with support testing for community acute findings?** | Sorry I did not hear the last part |
| Okay, I said can you share your experience during supervision of how the Truenat machine performed in the field in combination with PSD and support testing for community acute case findings? Have you ever had this experience or has anybody had this experience of going to the field with Truenat and then the PDS? | Yes. We usually take Truenat to the field during outreaches especially when IHVN are doing outreaches because on the field as we know, it requires minimal samples are tested for samples that are DSTB the patients are told their results immediately and we have not had any RIF case on the field; it is usually DS that we normally have most times when the machine is taken to outreaches. |
| What went well on such instances when you went out on community outreaches case finding? What and what went well and what and what did not go well? We are comparing in terms of Truenat and PDS? | So, for the Truenat, it went well because there was no power interruption and it was just at point of care samples were collected and the result tested are coming out at once. There is no instances of looking for logistics arrangement to shift samples to the lab before it can be tested. So, it went very well. |
| So what I’m saying is are there things that went very well and things that did not go very well? Just share such experiences with us. | All went very, nothing that I can say did not go very well. |
| Okay, thank you. So, number 6 are you still here with us? | Yes, I can hear you. |
| So can you share your experience with us during your supervision of how the Truenat machine respond in the field in combination with TBS CAD with support --- for community acute case finding? What went well; what did not go down well? | Okay. The truth is that in my state we have never gone out with any of the machine to the field for testing during outreaches. What we do in my state is that we go out for outreaches and the samples are brought to the different labs for testing. We have never gone out with the machines from the various labs to the field. We have never move them out. |
|  | The things that went well include the fact that the Truenat machine can be utilized in resource limited settings like PHCs, it does not require AC and it could be moved around easily.  The things that did not go well are that same person driving the wheel, collects sample, test and document. In some instances, the reagents required for testing are insufficient and there may be need for power to charge the machine. |
| Okay. Is there anybody here who have had a field experience with regards to this question? | RESPONSE: |
| I just got signal that this discussion will end in ten minutes. I’m not sure this one is up to 45 minutes. We’ve just had about 30 minutes. Okay continue. | RESPONSE: From my state, experience in the field: we have two keke given to us from the National. We use the keke by putting the Truenat machine inside the keke and go round during outreaches, collecting samples for testing. |
|  | Truenat machine is small and easily carried about in my state of operation. There are Wellness on Keke (WOK). Truenat machine is carried inside this WOK for active case finding thus test is possible during outreaches, community screening in combination with the digital X-ray and with this approach, a lot of work is done in the field.  After sample collection, testing is done immediately without delay, testing always go well the only problem is when the battery runs down and must require recharging. |
| I: We are having very serious issues here with hearing you and it is important that we share this your experience with us. So I will be pleased if you can make jotting of the question and send to me. I will appreciate it because from all your contribution the network has not been so wonderful to us. I am talking to the last speaker, I hope she heard me?  I: Okay, I will get back to her. It means that we may still need to come back because we have not gone half way. I don’t know how fast we can be again but let me go back to number 1. Okay has anybody has experience with community outreaches in using Truenat? Any other person? Has any other person has that experience? |  |
| I: Has any other person had such experience of using Truenat for acute case findings? Okay so I go to the sixth question. We will still come back to it if there is still need. I wonder how they are computing the timing now because they have given warning again. I am going back to number 1: **can you tell us one thing you consider a major advantage and a major disadvantage on the use of the Truenat machine for TB testing? Number 1 we are back to you. Number 1 we are back to you.** | Can you hear me? |
|  |  |
| Yes we can hear you. Go on. | Okay, one of the major advantages of Truenat is that as I said before, it is rugged, it is independent of AC- whether there is AC or no AC you can use it. Another one is that it differentiates between MTB detected positive and RIF resistance. It can tell you whether the sample detected is positive for TB or RIF resistance that is another advantage.  The major disadvantage is because of the number of modules that it has; at a time because it has two modules meaning we have to run only two samples at a time. Another disadvantage of this one is for instance if you detect TB and you want to run a test for RIF resistance, you will have to process the sample again. Can you hear me? |
| Number 4: can you tell us one thing you consider a major advantage and a major disadvantage? Number 4. Check if you are muted and unmute yourself, number 4. Okay number 5? | Yes number 5 can you hear me? |
| Yes, just tell us one major advantage and one major disadvantage of Truenat machine for TB testing? | The major advantage is that they require minimal infrastructure.  The major disadvantage of this technology is apart from sputum samples, you cannot use other samples to test for TB like stool. |
| I: Yes. You know there were disruptions. You know nobody will remember all these things off hand; that is why we are doing the recording; it is not clear. There is need to submit your contribution and this is very important for number 3 because all her good points were not picked and it is important we have them for our report. Number 3 I hope you are hearing and you will oblige us? | As you advised we can send it to you through WhatsApp |
| Yes but we can continue because we need to finish up with this; we still have a lot to do. We pause for a while because it will help us update whatever we are doing. Network comes in and disrupt us like what number 5 was saying now about the advantage, I did not even pick it. | But I am still on the line. I said the major advantage I consider is that Truenat requires a limited infrastructure- you need no AC and so many things that GeneXpert needs. And the major disadvantage is that you cannot test for with any other sample apart from sputum. |
|  | There’s down time due to lack of reagent with the attendant service disruptions. Some of the repairs could be done in Abuja and sometimes it may require that the machine be replaced. |
| Number 5 just finish up what you were saying let’s get one or two more views and we move to the next number. Hello number 5? Your microphone is not visible. Okay number 7 can you continue? Can you answer that question: one major advantage and one major disadvantage of the Truenat machine for TB testing? | This is number 7. One major advantage is that it doesn’t need AC. The requirement for processing is very minimal. It can work in rural area where there is no regular electricity supply. Then one major disadvantage is that it can only process sputum samples like in this month of May, there is going to be childhood TB week and this Truenat machine will not be used because majority of the sample that will be collected from these children will be stool for testing TB and Truenat machine cannot process stool samples. So it is a major disadvantage. It can only process sputum samples. It cannot be used to run any extra-pulmonary samples. This should be looked into. Thank you. |
| Thank you very much for this explanation. Number 2 can you finish up on that so that we can go to the next question? One major advantage and one major disadvantage of the Truenat machine for TB testing. | The major advantage is that Truenat machine is rugged and it can be used anytime anywhere with little infrastructure. Then the disadvantage is that it can only process two samples at a time. Like the one we have right now in the country, it can only process two samples at a time. unlike when we will have the quadro machine that can process four samples at a time, it will no longer be a disadvantage |
| Okay, thank you. Number 3 can you give us one advantage and one disadvantage of Truenat machine for TB testing. Number 3 are you there. Remember we will need extract from you because we are missing all your important contribution because of network. Number 3 can you give us this or we join it with what you are going to give us at the end/ number 3 ma? | Hello, good afternoon |
|  | The size of the Truenat machine is an advantage in terms of ease of carrying, it is mobile but a major disadvantage is that it cannot be used to test stool which aids TB diagnosis in children that cannot produce sputum. |
| Number 1: I’m asking you number 7 question, number 6 I am not seeing the sign of her microphone so can you tell us the common challenge or problem that have been reported to you from the sites implementing the Truenat? | Okay. The major challenge from site which has been reported to me is with regards to samples. They are not getting plenty samples in that area. So, we have sensitized and in this sensitization, we have moved round places like other PHCs to see that they mobilize --- for the Truenat machine yet no result. So that is the major challenge here in my state especially in that area where our Truenat machine is located. So we have tried to mobilize other health facilities to send samples to the Truenat machine; that one we have taken it upon ourselves to use the Truenat machine to go to communities and sensitize them. So this is the biggest challenge we have in my state. The samples are not enough for us; we need samples to run but the samples are not forth coming, so we are going to use active case search in the communities together with keke (the tricycle) (in collaboration with KNCV) and together with the Truenat, we will see if we can get samples and test as many as possible for TB using the Truenat machine. So that is our desire. |
| So is it affecting or is there any downtime? This problem you are having, is it causing any down time? | It is not causing any downtime because the problem is not with the machine |
| Okay. All you are saying is that you are not having enough samples? | Yes. If you are talking of downtime, last quarter we had issue of what we called cartridge stock out for the machine, so we did not work very well. The workload was low because we had a breakdown- we ran out of cartridge for either one month or so. And our work was down. So our major challenge is sample- how to mobilize samples for that Truenat site and that is what they are complaining. That is why we want to collaborate with KNCV to use keke in the community together with Truenat and the TB lamp and make sure they get samples for that site. Thank you. |
| Okay, thank you. Number 6 I’m sure you are back fully now. Can you tell us the common challenges. | I just joined and so I didn’t hear what you said |
| But can you hear me now? | Yes, I can hear you well now |
| Okay I am coming with the question now. So, can you tell us about the common challenges or problems that have been reported to you from sites implementing the Truenat? | The common challenges? |
| Yes. | Okay. The challenges that they reported sometime last year is a report on stock out. The cartridges were not there. That was what they reported early this year which I think we have supplied. But before now, in one of the sites, it was manpower that was their major challenge. They were complaining of workload for that particular site. That machine has also been breaking down and we have been struggling on who to put there because of human resource constraints. You know that the machine does not use any kind of paper, the paper they are using they were not able to buy so they cannot print their results. And in writing, we have found out that writing out their result has been a challenge and that site has been giving us a lot of issues because of documentation problems and result writing. So we are of the opinion that instead if they have a paper that they should rather print out the result, we get the result and it will be better. We have been doing a kind of advocacy to the management to see how they can help them meet up with these challenges that they had which caused some step down; like bringing ‘Youth Corpers’ to help them for the workload in order to meet up. Then apart from that, I know the workload there is much but like I said commitment to duty is the key. The focal person in charge is very dedicated so we don’t have an issue with that site. Apart from these little challenges, which we are able to sort it out. |
| Does it mean there has not been any report on the functionality of the machine itself? | Yes, there have. There is a HOD in the lab that takes control of that. |
| What I’m saying is has there been any report that came that the machine is not functioning or that this is not working? Has there been such report? | No, the machines have never stopped working |
| Okay | It was only the one of our machines that broke down; and it was taken to Abuja from where it was taken to India. That was sometime last year- in November or thereabout. |
| Okay. So,all those ones are small issues? | Yes |
| Number 7 I will continue with the 7^th^ question. Can you tell us the common challenges or problems that have been reported to you from sites implementing the Truenat? Hello number 7 | One of the problems is that I discovered that more errors and invalid results have been generated from Truenat when compared with GeneXpert. Then apart from that, we do have occasional breakdown of the machine and we will not be able to test samples with the machine. Then last quarter there was total stock-out of Truenat reagents so we couldn’t run samples with the Truenat machine throughout the state. We had to move our samples from the Truenat sites to GeneXpert sites. So it is the major challenge that we have. Like I said we have more errors and invalid results compared with GeneXpert. Then it took a big sensitization and we also had to take our stand that TBLSs and Riders for Health that were in those areas where Truenat machines were located should take their samples to Truenat sites and not GeneXpert sites because some of them seem to prefer taking their samples to GeneXpert sites because of the turnaround time. So we had to enforce it on them that since Truenat has been supplied to this site, they should be ready to utilize it for their diagnosis. So that is why we need the quarto machine instead of the duo machine so that more samples can be tested and the turnaround time for the machine can be reduced. Thank you. |
| Okay thank you. Number 2, Can you tell us about the common challenges or problems that have been reported to you from sites implementing the Truenat? | Yes sir, the question again sir? |
| I: I said Sir, can you tell us about the common challenges or problems that have been reported to you from sites implementing the Truenat? | The common challenges are firstly, human resource. We have two of our sites that are complaining of overload of work and they need additional human resource. Even though the staff on duty are trying their best to mop up samples, they will require more hands. Secondly, another challenge is that occasionally they have stock-out of reagents in the state but presently there is nothing like that though there was a time they had total stock out and during that period, no job was done. |
| That means it affected the output then? | Yes. |
| But now the stock-out is over? | It’s over; all the sites are stocked now. |
|  | Lack of support to fuel available generator, incessant transfer of staff without replacement There was downtime for more than two weeks, it reduced the number of test and this also affected active case finding in the LGA. The output was reduced. |
| Can somebody with a different opinion from what they others have said tell us about this- the challenges or problems that have been reported to you? Anybody? |  |
| So, we can move on to the next question. Does anybody has a different impression? No 5 sorry, I will definitely call you or you just prepare your mind. Your opinions are important that is why we need them. But I know the network has been very bad but bear that in mind |  |
| So, any other information on that question number 7? Anybody | RESPONSE: Yes |
| Okay no 4 I think you are having network issues. Let us not kill our time. I will definitely call you, we will request of you to supply us your answer. I can see that the network is bad where you are. Number – do you have something to say otherwise let me move to the next question? |  |
| Okay I will move to the 8^th^ question. Number 6 are you there? I need to be confirming. | Yes sir. |
| Okay. **Thank you ma. What has been your experience with the use of Truenat for service providers since implementation? The service providers- Truenat service providers what have been your experience with them? They repairs, and maintenance, Do** they respond on time to replace a new specimen? | Yes, they do |
| So, you can give them a pass mark? Are you giving them a pass mark? | Yes, I can score them very well |
| Can you give us an instance? | Yes like when they were called concerning one of the machines in my state that broke down, it didn’t take up to a week. They came, checked to see if it was what they will be able to resolve. So when they saw that they will not be able to resolve it, they moved it to Abuja and there at Abuja, they found out that it was beyond them and they took it to India. So because of the fact that it was what was going to take time that was why the machine took long. But I am saying that I’m giving them a pass mark because they are always coming down almost every time to check on the machine; unlike GeneXpert that they don’t do that too frequent. That is why I am saying that I am giving them pass mark. Even this week, they have come for one of the machines in my state, so they are always there to check one thing or the other. So that is why I am giving them pass mark, they are doing very well and they should continue with that. |
| Thank you very much. Number 2 the same question: What has been your experience with the use of Truenat for service providers since implementation? The service providers- Truenat service providers what have been your experience with them? The repairs and maintenance, Do they respond on time to replace a new specimen? | Well I will say for all those sites where there was challenges---.there were times when there was a total breakdown with the machine So it was transported to Abuja and from Abuja it was taken to India for repair and that took a whole month and it affected service delivery but in all there is still a good response from them. |
| Okay are you through? Then number 1 just give me your response to that question so that we can move to the next number. What has been your experience with Truenat support providers since implementation? How well do they response to repairs and replacement? | Actually, they responded fast though I have never had issue with the Truenat machine but in case of stock-out of cartridge as I said before, they responded fast. As soon as we reach out to them that we are out of stock and within one week, they supplied us with enough cartridge to continue with our work. So they are responding well and on time without delay. |
|  | Truenat service providers are doing well as far as I know. Even though none of them reside in my state of operation, whenever there is challenge, need for repairs or replacement, they are very responsive as soon as a call is made, they respond within a few days.  In some instances there could be trouble shooting by the user, they, (the Truenat service support providers) respond by telling us what to do. However, once it involves moving the machine out of the facility, it takes 5-7days for the machine to be returned to us. |
|  |  |
| Okay. So we will continue; I’m still happy that some people having network issues are still with us. We need submitted document from them. So I’m appealing with us so that we can come to an end. Number 6 I think you have been very good with your internet and so we appreciate that. **So I will go to the 9^th^ question. What are your thoughts about the reliability of Truenat test result compared to other diagnostic test? Explain. Number 6 ma?** | The reliability of the Truenat test I think I will say it is just okay. I will say it is the same thing when compared to other test like GeneXpert; it is very reliable when compared to GeneXpert because it is the same way that GeneXpert will test for MTB detected and at the same time for the RIF Resistance, that is the same way molecular diagnosis that is from Truenat is done. And it is reliable. And for as long as you are doing quality control, you don’t have any reason to doubt the result, once we do quality control and it passes and we do our monthly/weekly maintenance as at when due. So as far as I am concerned and to the best of my ability, the Truenat tests, they are as reliable as GeneXpert tests. They are very reliable. |
| Thank you. Number 7 | To me, the results are very reliable. When it comes to quality management what I did apart from the quality control of the machine before running the samples; some samples were taken to Truenat sites, same samples were also taken to GeneXpert sites just to be very sure that we are getting good results. And the two came out with the same result so showing that these Truenat results are reliable and that there is no reason to doubt results obtained from the Truenat machines. So with that quality control-the external quality control done on the samples taking to the Truenat sites being the same samples also taken to GeneXpert sites and obtaining the same result. This shows that they results are highly reliable. Thank you. |
|  | Other countries have carried out the sensitivity and specificity comparison testing to GeneXpert and its on record that the performance of the Truenat MTB, MTB RIF DX show comparable accuracy to the Xpert MTB, MTB RIF, but personally I haven’t done that. In any case I believe that report that they are comparable |
| Thank you very much ma. Number 1, what are your thoughts about the reliability of Truenat test result compared to other diagnostic test? | The Truenat test always give a reliable result. We usually conduct quality control on our machine to make sure the machine is working very well and that the machine is giving a reliable result. This quality control is very important. In whatsoever investigation you are going to do, you must add your quality control so that you will be sure that the test you are doing, the results are reliable. So there are some external people that come for us to check and all our samples that show positive MTB- if you take them to other tests like GeneXpert or even the Truenat, you still get a positive result. So the results of Truenat are very reliable since any result that is released when taken to any part of the world, it will still give the people the same result. That is how reliable it is. It is very reliable. |
| Number 1 | Sir |
| Okay it’s number 1 that just spoke | Yes sir |
| number 2. | Okay sir |
| We are talking about the reliability of Truenat test results. | Yes, it is very reliable. |
| Just explain with one instance on why you stand on that | We have tried testing with another machine and |
| (cuts in): Okay let me go to the next question; they are already warning us: so that even if we come back we finish up because we just have three questions to go so let’s do justice to all of them.  Number 2 I’m starting with you on a new question**. Based on your interaction with other healthcare workers like ---, those focal persons and nurses providing TB care, how confident are they with the results generated from the Truenat sites?** | They are very confident because we gave them information before the machine was installed and immediately after the machine was installed, we had meetings with them also, we called them to see the machine, told them what it can do and how accurate the results are. So, they are very comfortable with it. |
| Okay, thank you. We are being prompted. I am sure when we come back, we will be able to complete this section. Please I thank you for the perseverance so far. I didn’t know the cut waves will be there.  I: Number 6 let me come back to you. Based on your interactions with other health care workers and service providers and all that are providing TB care, how confident are they with the result generated by Truenat? | They are very confident with results from the Truenat machine because before Truenat was installed, and immediately after the installation, there was a sensitization meeting and training that took place among all the general health care workers. They did sensitization, they did training for them to let them know what and what the Truenat machine is able to do. Based on that, they had information about the machine, the type of samples to send and all that. Just like we did when GeneXpert was coming on board and based on that, we did the same thing with the Truenat as soon as it came on board too. And as soon as they have seen the result coming in like the RIF Resistance cases, MTB cases, they knew that it is one and the same, so they are very confident and very okay with it. |
| Thank you ma. Number 7 | Like some of these officers |
| Please can we be more patient; we just have two more questions and we will go. Number 7 please go ahead. | Like some officers when a sample is brought and the result is negative, some of them because of their experiences will doubt the result. What they normally do is to collect another sample from the same patient and take it to GeneXpert site. Taking it to the GeneXpert site, they are getting the same result and this has helped them about the reliability of Truenat results. Then instead of them carrying their samples to GeneXpert sites, they will now take it to Truenat site that is very close to them because they are now at home with the Truenat result. Having gotten the same result, they now trust the results from the Truenat machine. Thank you. |
| Thank you ma. Number 1. I am sure we may be cut off very soon. I want to thank you for your perseverance. We just have two more questions. We will go and come back and complete it. But number 1, let’s finish up on this. Based on your interaction with health care workers, technicians and other service providers providing TB service, how confident are they with the Truenat test? | They are very confident of the results from Truenat machine. They are confident because sometimes even if the Truenat does not give them the result that they expected, they take the same samples to GeneXpert. And when they compare the result of Truenat with that of GeneXpert, and if they find out that the results are the same, they started trusting the results from the Truenat machine. In-fact, this approach was what made them to be confident of the results from the Truenat machine, because of the comparison of the results with what was obtained from the GeneXpert and seeing that they are the same. So they regard results from Truenat as being reliable. |
|  | The result from Truenat machine is very reliable and this gives us confidence. However for any molecular test to produce a reliable result the staff must follow the standard operating procedure strictly.  The manufacturer of Truenat machine, (Molbido) developed standard operating procedure which guides anyone using Truenat machine for testing of TB just like GeneXpert has its own SOP and once the SOP is followed the result is always reliable just like that of GeneXpert.  In my state of operation, the results obtained from Truenat are well accepted since they were informed before-hand that it works like GeneXpert. Based on this, they do not doubt the results from Truenat, they consider the results very reliable. If ‘Quadro’ Truenat machines are supplied they healthcare workers will see them as being the same as GeneXpert machines. |
|  | Yes, they do, as long as it is one of the W.H.O approved diagnostics, its certain that it has been validated and assured to be fit to use because it met the required criteria for being one of the point of care diagnostics. |
| **Based on your context, Can you think of factors that could act as barriers to the scale-up of the effective use of Truenat Tests for TB diagnosis to more LGAs/facilities?**  **a). (PROMPT: Is there anything missing? Is there anything/factor that could affect carrying out the Truenat tests? (Number 2)** | One of the factors is human resource. Most of our health facilities don’t have enough health workers for the work they should be performing. |
| We just have two more questions to go; I think number 2 was saying answering before we were cut off. Have you finished sir? | Yes, I have finished. |
|  | Staff attrition is a serious problem as most of our staff are either volunteers or ad-hoc and the permanent staff are retiring and most times without any form of replacement |
|  | Laboratory consummables are not adequate as Truenat Testing requires more hand gloves, disinfectant, coded waste bags. Again, Decontaminants are not readily available most times. |
| Okay. Number 6 have you joined?  Okay number 6 and 7 have joined. That is very good. Number 6 can you tell us factors that can act as barriers to the scale up of the effective use of Truenat test for TB diagnosis to more other Local Government Areas or health facilities? | I think the major factors that will act as barriers are like my colleague has said, shortage of health workers. Only, if they should employ more health workers in our health facilities. If they can get personnel. Because since the Truenat machine is something one can easily train somebody on. It is something that is easy to learn. Once they can get personnel to be trained and they can get space to keep the machine that will be good. Most of the health facilities complain of space- getting a space even if it is a small space is a problem as the buildings have been long built and occupied. So I think personnel and space are the problems. Once they get personnel and get space, we are good to go. For me those are the two major barriers I think if handled, we are good to go. |
| Number 6, still on you, is there anything that could have helped carrying out Truenat test? We are now talking about expansion. So you are talking about space, could these things affect them in carrying out their work? | Yes, once we can get spaces, then get personnel; then there won’t be any problem. Then more Truenat can come in because there must be places to put them and there must be people to test. That is why I see them as barriers. To bring the machines, there must be where to keep them; I don’t know for other states, but I think it is a general issue: many people have retired and there is no employment being done. You will see a work that ten people should be doing, one person will be doing because people have retired. People retired they don’t employ. Even if they don’t employ more, they can employ ad-hoc staff, train them and they are good to go. But the challenge with ad-hoc staff is that so many of them they don’t last, if they see a better work, they can leave this one and go to that better work. That is just the disadvantage of it. But meanwhile they can do it while we look for other ones. So those are the two things- personnel and space. |
| Thank you very much. Number 7 | I want to add to the area of personnel; I want to add irregular supply of reagents or interrupted supply of reagents. Sometimes there will be time where samples will be brought to the facility and there will be no reagent to run the samples. Then apart from that again, there is need for back-up especially that these Truenat machines are located in rural areas where there is no regular supply of electricity. In a place where there is no electricity supply for maybe three to five days, the battery would have run down. So to run the samples will become difficult. So there is the need for back up no matter how small; whereby the batteries can be charged. Then there is the need for the maintenance of the machine before breakdown. Maintenance should be put into consideration. They should not be waiting for when they will be called that the machine has broken down before coming for maintenance. There should be repair and regular maintenance of the machine. Thank you. |
| Thank you ma. Number 1 sir. | Yes, I want to go with them with regards to personnel. But there is another issue like in my state, we have security problems. Some local government areas in my state (about five or six) with high TB burden and high sample load have security problems and because of that we cannot install Truenat machines in those areas. So tackling the problem of insecurity will be very important in my state. So I think that the security issue should be improved upon so that so that some Truenat machines or the new ones that will come can be placed in those Local Government Areas where the TB burden is high but presently have security problems. Thank you. |
|  | I don’t see any barrier but we need a backup power as most LGAs in my state are not connected to national grid. Also there is need to provide storage facilty for samples incase quatro will not be supplied, so that excess samples can be well preserved. |
|  |  |
| **Thank you very much. I think this is the last question. I will start with you number 2; can you think of factors that can facilitate the scale up on the use of Truenat to other Local Government areas or facilities? Please give an explanation. Number 2 sir.** | Sorry number 2, I was a little bit distracted, can you repeat the question? |
| Yes, I will come again with the question, I think this is the last question: I said can you think of factors that can facilitate the scale up of the use of Truenat to other Local Government Areas or health facilities? | Yes, if we can get scale up with facility improved in the laboratory- like most of our laboratory are outdated and there is need for refurbishing. Some don’t have even have good ventilation; so refurbishing and employing people to work will be important in getting people to go and work in that facility. They should also improve working condition. |
| Yes. | Thank you. |
| Number 6, can you think of factors that can facilitate the scale up of the use of Trunat to other health facilities and LGAs? | Yes. employment of health workers, good working conditions, minimal infrastructure- by the time they will provide the lab no matter how small and employ and then provide the machine. Then there can be a scale up. Once they provide these things, scale up will come. |
| Because anytime they provide the machine obviously they can tell you on the use of it? | Yes. Once they provide, they train. |
|  | Provide source of light, good sample referral network, regular supply of personal protective equipment and lab consumables for provision of uninterrupted service, prompt release of results (short TAT)  Yes, motivation for staff carrying out testing as is being done for staff working with GeneXpert. This is important for maximum productivity. |
|  | .  In some hard-to-reach areas; some LGAs are still running TB Smear microscopy even for diagnosis of TB, because of difficult terrain to move samples to other molecular test sites. This approach will make it difficult to eradicate TB if we do not know whether we are dealing with drug susceptible or drug-resistant TB. This approach will obviously make the scale of Truenat necessary and urgent |
| Okay. Thank you. Number 7 | In addition to what have been said, I think the first thing is that it should be integrated in the facility. It shouldn’t be seen as a different machine. In the department, it should be integrated in the department as part of their tool and everybody in the department should be made to participate in using the machine. Meaning that everybody in the department should be trained on the use of Truenat machine. Then apart from that as it has been said, the Truenat machine should be changed to a four-module machine so that the TAT will be reduced  Then the prompt maintenance as it has been said, prompt replacement of faulty machines. Those are the things I think that can help- the factors that can help to improve the use of the machine. |
| Thank you very much. Number 1 sir | Yes, they have said it all. You know if you want to scale up Truenat machine to other Local Government, you have to consider the facility first or the lab. Do they have the things needed for this machine to take off in the facility- because if you go to some facilities; like some primary health centers, you will see bench so bad, or you will not see any bench in that facility or in that lab. So, these little things are supposed to be made available before scale up of this Truenat machine to other Local Government so that at least if you are taking this thing to Local Government, you make sure that the lab or facility they are taking the machine to be in order. Like the chairs they will sit down in running the rest. Because in some of our places here, you will just go there and even the bench to sit down you will not see in the facility. So, you cannot put the machine there. At least you are supposed to have a working bench where you will be able to keep the machine and start using it. So, it is very important to consider little things that the machine need. Also, the security of that health facility- you have to consider it also because you cannot put Truenat where it will be broken and stolen. So, all these little things will have to consider them before scaling up Truenat. And you also have to consider the population- where are having high TB burden so that we now scale up the Truenat to those areas because if you just carry it to anywhere, you will start having issues of samples. You will not be getting what you want and this is based on my experience here in my state. Because where we keep our machines to, in fact samples are not forth coming. So, we have to look at the location very well if there are a lot of samples coming to that Local Government before we can place the machine there. So, when we want our scaling to be very good, we have to consider all these few things. Thank you. |
| Thank you very much. I think this is the last question. I don’t know if anybody has any other comment to make that you consider very important to this discussion but was not included in any of the question that we have passed? | Yes, a comment. I am number 7. My comment is about infection control. If they should put into consideration in Local Government where we have Truenat machines, infection control system |
| We are having very serious network issues. We have been missing your points, I think the network has just suddenly gone bad | Can you hear me now? |
|  | The following need to be put in place. A space for the Truenat machine, access to the health facility, power to charge the Truenat battery, printing paper for the printing of test results and introduce quadro Truenat machines. The quadro machine will increase the confidence of healthcare workers in the results obtained as they will see the machine the same way they see a GeneXpert machine. |
| Yes, I can hear you now. | What I am saying is that infection control system in sites where Truenat machines are being placed and majority of them are primary health centers where there is nothing like infection control system. So while bringing more Truenat machines into states, infection control system must be put in place, they need bags and other things for infection control. This should be part or be put in the plan. Thank you very much |
| Thank you very much for that suggestion. Any other person with any other opinion? | Yes, I am number 1. Are you hearing me? |
| Yes. | There is need again for storage system- that is refrigerator. Yes, refrigerator is very important for the lab. Because you will go to some primary health care, they don’t have refrigerator. For instance, if they get 20 samples and if they cannot be able to run those samples in a day, where will they store them? Do you understand? |
| Yes sir. | So, if they have refrigerator to keep some samples that they cannot be able to process on that day (so that as soon as you are not able to finish your processing in a day), you put some in the refrigerator for the next day to continue. So storage system is very important whenever we are scaling this Truenat machine. It is very important. Let us consider storage system- refrigerator. Thank you. |
| Thank you very much. Any other comment? | Yes, number 4. |
| Okay. | In my state we have the issue of personnel protecting (PPE). These things are very essential for protecting the lives of people that are doing the job. At present there is inadequate supply of PPE materials especially in our TB laboratory. When PPE materials are not supplied, we put our staff at the risk of getting infected. We already know that we don’t have enough staff, so the few that are working we should not endanger their lives. So when we are thinking of scaling up, we should also think of consumables like PPE that will make life easy for people working in the lab. That is all I have to say. |
|  | What is missing in the Truenat machine is its inability to test extra-pulmonary samples hence children that cannot produce sputum but are infected by TB have the challenge of prompt diagnosis. Also, without light it is not possible to run any test if the battery is dead. |
| Thank you very much. Thank you for that suggestion. Any other comment? | Yes, I have. Just like my colleagues have said, I will just say that accessibility of the facility is very important. It should be located in a place where the facility can easily be accessible because if the facility is not accessible, that will pose a challenge to samples being taken there frequently. Very importantly, my colleague has mentioned regular supply of PPE and consumables. This is very key especially if the Truenat machine is installed in hard-to-reach area. You know that such areas you don’t just go there every day; so they should be given enough PPE and consumables to work with. And also, renovation- minor renovation should be carried out in the lab. You know, after assessment of any health facility there should be a time frame for the identified gaps to be rectified before taking the machine for installation for the smooth running of the machine. Thank you. |
| Thank you very much. Any other contribution?  Okay, so I can’t thank you enough for this. I think I am very pleased to have started this focus group discussion with the state quality assurance officers because they have proven to be champions. It is very commendable so I thank you all for participating. | (a response)Thank you sir. |
| And happy workers day to all of us and I am happy many people talked about the workers welfare. Thank you everyone. | All- Thank you sir. |

**FGD 4**

We start

Okay, number one

**Question 1:**

**What has been your experience so far while using the Truenat test for TB diagnosis in your facility or Local Government area?**

**Number 1**

The experience has been good. You know, departing from the AFB slides that we have been doing, the Truenat has been fast and reliable. We have been able to obtain accurate result. More so, it has control inclusive. So it has been okay.

**Number 2**

The experience has been very fantastic and fast. It affects everything effectively. In terms of case finding, it has increased our case finding in the LGA. Considering the transportation of our samples to the GeneXpert side which is very far from us, we are able to access Truenat in all the facilities. It has been very fantastic. In terms of workload also; because we generated a lot of samples in our LGA, being a budding TB facility. So it reduced workload, and affects everything positively.

**Number 3**

It increased our case finding - the result is out very early, not wasting time. If I take sample to the lab today, at times the result can come out that day or the next day and that means that if the test is positive, the patient will start treatment immediately.

**Number 4**

It has been wonderful having Truenat in my LGA. Formerly we had only one GeneXpert machine in my LGA and it's for four modules. What happened is that by taking all the samples to GeneXpert, sometimes there will be a lot of backlog. But Truenat machine made it easy and faster for us to get results. And be a source of support for the GeneXpert, because a lot of samples were being taken to the Gene Xpert. Now for those private hospitals their samples are now taken to Truenat machine; Truenat is faster, within an hour, and it also detects MTB and RIF-resistance also. It makes it easier now for us to get our results as easily and fast as possible. So this Truenat in my LGA really helps a lot.

**Number 5**

Thank you very much. Good evening all.

It has made my work very easy compared to before. Before, in our LGA, we don't have any Gene Xpert machine, not until the day they brought Truenat in my LGA. In fact, it makes my case finding very easy, since Truenat can detect MTB and RIF Resistance. It's very good.

**Question 2:**

**How has your practice (TB testing, case finding, and service delivery) been affected by introduction of the new Truenat MTB, RIF machines?**

**Number 5**

It affects the work.

You know Truenat only picks two samples at once; so in that aspect it may be poor, but the result comes out at any time you mix it; so it makes case finding very easy.

**Interviewer**

So it affects case finding?

**Number 5**

It does affect case finding

**Interviewer**

Okay. So has the case finding in your facility been high now or it has decreased?

**Number 5**

Truenat has improved our case finding. Last month I can boost of having 30 patients that are on treatment, compared to before that we use to transport samples from our local government area to another local government area. Since the introduction of the Truenat machine, things have been very easy for us.

**Question**

So what about your own workload as a person? Has Truenat increased it or decreased it?

**Number 5**

It increased my workload

**Question**

How did it do that?

**Number 5**

Because of the cases, comparing to when I'm not having cases. Though it's not long I joined the TB supervisors, anyway. But as at the 2nd quarter 2022 that I joined the TB forum, we were not having enough Presumptive cases, but it was that same 2022, when they introduced Truenat machine in my LGA, case finding increased. So it makes me have more workload, because of the increase in the number of patients.

**Question**

Okay. Thank you.

Over to Number 2

**Number 2**

Truenat affected my case finding positively. Compared to some previous quarters, it increased my case finding by almost 30%. In terms of workload, it reduced the workload at our Gene Xpert site because it serves as support to our GeneXpert site. It also reduced the turnaround time for results of samples to be received from the lab. During our outreach time; the time we do outreaches, we normally take samples to far far away Gene Xpert sites because of the high number of samples. But since this Truenat is very mobile, we do go along with it. So we are having instant diagnosis and instant services rendered.

**Number 2**

Yes, Truenat increases case finding, reduces our turnaround time for the samples to be out. It reduces the burden that we're having at the outreaches

**Question**

Okay. Now what about your own personal workload? How has Truenat affected it?

**Number 2**

It reduces my workload

**Question**

How, sir? Just give me an instance

**Number 2**

For instance, considering we're having a huge amount of samples... People will be disturbing us over the phone for their results, because the turnaround time for the GeneXpert result to be out is 2-3 weeks, even up to a month. But now the burden of calling us and disturbing us over the result has been reduced. That's how it affects me personally; because considering the person that is being requested to do the GeneXpert test, and the results are not out in time; me personally, I consider if I'm the patient, how will I feel? 2, 3, or 4 weeks for results to be out; it takes a long time, I would have found another option. But with this Truenat we're having instant diagnosis and instant services if needed.

**Interviewer**

Thank you.

Number 4

**Number 4**

Okay. Actually, in my LGA, both private and public facilities take all their samples to GeneXpert site at the General hospital, and this caused a lot of workload to the Gene Xpert site. So when the Truenat was introduced, all the private facilities... Because the modules for the expert is four, but the Truenat is just two that it can run at a time. For Truenat now, all the private facilities, since they don't have as much patient as the public facilities, they should be taking their samples to Truenat machine, and this makes it faster. The other time, when they took everything to Gene Xpert, before the result is out, sometimes it's late... Sometimes some patients would have gone. But since the Truenat was introduced, all my private facilities were being taken, and some of the public facility that their workload is not much were taken to the Truenat machine for the examination. This makes it faster - within an hour the result will be out. The turnaround time for the result is within a day - we have to collect it that day or the next day. This has resulted in more case finding in the private hospital than before.

Concerning the workload, it reduce the workload of the Gene Xpert site, because the private hospital were accepting Truenat, which is also serving the purpose of Gene Xpert.

So, in my LGA, Truenat reduced the workload in the GeneXpert site and at the same time, the turnaround time for the Truenat is faster than the Gene Xpert. And you know, if the Truenat machine, even if there's no light, it uses battery. This makes it easier and faster.

**Interviewer**

Okay. Thank you.

Number 3

**Number 3**

In my facility we're enjoying the Truenat machine very well, because before we don't have any Gene Xpert. We take our samples to a different LGA Before we get our results it takes time, but now we have Truenat machine and the results come out almost immediately.

**Interviewer**

Okay

**Number 3**

And it makes early diagnosis and treatment possible.

(Poor network)

So we don't have any problem now. It helps our case finding. The machine works faster.

The workload...

(Poor network)

**Interviewer**

Okay. Thank you.

I hope that as we progress, your network will improve so we can hear you very well.

Number 1, just respond to that question so we can move to the next question

**Number 1**

The Truenat has made our work very easy.

It has increased our case finding. Unlike what we used to have before, case finding has tremendously improved, because of the Truenat machine.

And the service delivery is excellent. Our turnaround time has improved drastically.

Then workload, I'll say that for the person that is handling the Truenat machine, there will be increased workload. Our workload has increased, positively anyway. Yes, positively, because people are appreciative of what we've been doing; so more samples are being channeled to our site but it is a positive workload anyway.

Thank you

**Interviewer**

Thank you very much.

Question 3: **Do you think the Truenat test for TB diagnosis has been successfully integrated into the TB diagnostic network? If you say yes or no, you give an explanation.**

**Number I**

My answer is yes. I think the acceptability of Truenat has improved. Many people prefer the Truenat diagnostic outfit more than going to the other traditional outlet, i.e. the Gene Xpert, because of the speed of receiving the results. This is also positive because that helps in patient treatment. So I think the acceptability has been high. I believe it has been integrated very well, and it's accepted very well. Now the GeneXpert is now like something to add up to it.

**Question**

Okay. But what about integration into the TB diagnostic network? Do you think it has been well integrated?

**Number I**

I think, in my area, yes. I will say yes.

**Question**

Okay. Why would you say yes? What is the evidence for you to say yes? Or what do you have in mind when you say yes?

**Number I**

From my own understanding of integration, I look at it as a proper channel from which results are being expected from. I think Truenat, after it has been introduced the acceptability has been high. And then the turnaround time, people are more in tune with these services. So I think the integration is okay.

**Interviewer**

Number 2

**Number 2**

I can say yes, Truenat has been fully integrated. For instance now, the R&R tools that we normally fill for the quarterly report, you can see that the column of Truenat has been added there, and also it has been accepted and put into the national guideline. That's why I can say it has been successfully integrated. Inside the national guideline, Truenat has been explained as a diagnostic tool for TB.

**Interviewer**

Okay. Thank you very much

Number 5

**Number 5**

If I may say, yes. Truenat machine has made work easy for presumptive and DR-TB. Then it has the high rate of RIF-resistant detection. So that is why. And it's very sensitive for detecting mTB sputum samples. So that is why my answer should be yes.

**Interviewer**

Okay

Number 4

**Number 4**

Yes. In all our reporting tools - case finding, sputum conversion - you will see Truenat. Any case being detected using the Truenat machine must be recorded. And even at the end of every month in my state, we report the total number of cases diagnosed with Truenat, those ones on treatment, and we also give account of the treatment outcome in our register. So everything about Truenat are integrated in our reporting tools. And at the same time, Truenat is also recommended by WHO, so to detect MTB and RIF Resistance Truenat is very good.

**Interviewer**

Okay. Thank you

Number 6

Yes, Truenat test for TB diagnosis has been successfully integrated into the TB diagnostic network. This is because the WHO has recommended it, the Global fund has approved of it and there is a column for Truenat in our Recording and Reporting tools and even our case summaries have Truenat included.

Number 3

**Number 3**

Yes, it has been successfully integrated because... (Poor network)

Even our summary tools, there's column for Truenat.

(Poor network)

**Interviewer**

Okay. Thank you.

**Question 4:**

**Practically what has been your experience with the use of Truenat machine for the evaluation of presumptive TB samples compared to other TB testing platforms that you have had experience with?**

Number 4

**Number 4**

**For the test** of presumptive and concerning the Truenat, it's good. Because even if any case is being presumptive, they take it to the Truenat machine, for the Truenat test. The result came out as fast as possible. And immediately it comes out as fast as possible, the patient is being enrolled on treatment. So it makes it easy. And I said earlier on, in my localization, I mean my state, it's mostly based on private facility... because the Truenat cannot take as many samples because it runs twos at a time; not like the Gene Xpert that runs fours. So mostly the private hospitals that have little cases that is not as public facility. Unless if in a Truenat, after collecting the samples from the private and they have some things again to do, so we can chip in from the public facility which is not supposed to be much. So in my state, it's being good in presumptive cases by detecting the... And take it to the Gene Xpert for diagnosis, and the result will come out as early as possible; there's no... It's good; it's a good experience. It's been good.

**Question**

Okay. So in terms of quality of results? I'm still with you... The turnaround time? How is input in terms of the number of tests?

**Number 4**

The turnaround time is faster - within an hour. Because when you move a sample, you know before you take a sample to the Truenat, to where it is... Let's say a day; by the next day they will receive the result. The turnaround is a day.

**Interviewer**

Okay. What about the quality of results?

**Number 4**

The result is okay. What GeneXpert did is what the Truenat is doing. For those that are running it, I think they have been trained to do it, and they are doing it well.

**Question**

What about the number of tests done at a time?

**Number 4**

At a time, it should be like 10-15. It's two they will do. The module is two according to this thing, but they take sample like 10-15. They run it in a day.

**Interviewer**

Okay. So they can run up to 10-15 samples in a day

**Number 4**

Yes

**Question**

When compared to Gene Xpert, how many samples could Gene Xpert run in a day?

**Number 4**

You know GeneXpert has four modules, and the four modules runs for two hours at a time. When they put the four samples on it, within two hours the four is out. If nothing happened, the four is out within two hours. Another two hours, another set will be out. In a day for the Gene Xpert, sometimes they may run up to 20 or 30. Some Gene Xperts do have up to 10 or 16 modules, but the one in my LGA just has only four. And the Truenat is supporting the GeneXpert.

**Interviewer**

Okay.

Number I

**Number 1**

Thank you. Practically, it is user-friendly. Even going back to when the training was done and I had to do step-down for my staff that didn't attend the training; when I did it the rate at which they learnt it was very fast. And when we gave them the test to do it, they were able to catch it very fast. So it's user friendly.

As per the quality of the results, first of all it is faster compared to GeneXpert. Truenat is fast. The accuracy is reliable because it is comparable to what we get from the Gene Xpert.

Then the turnaround time drastically reduced compared to what we have before.

(Noise at the background)

**Interviewer**

Okay.

Continue Number 2

**Number 2**

Practically it has been very very wonderful with the use of Truenat in evaluating our presumptives. If I want to compare it with the number we used to report quarterly, Truenat has contributed up to 30%.

In terms of quality of the result, I cannot say it's 100%. There might be error a times, because comparing the nature of the samples they normally brought to the Gene Xpert site, there's no sample taken to the GeneXpert site that it will not run, provided that it is not bloodstained. But with this Truenat, it rejects some samples. I have not known anything that causes that, but it may be error from the machine or any other thing. Or it might be external error or the quality of the sample. But there are some samples that are of good quality, that I do evaluate myself, but Truenat will reject it. That's what I can say in terms of quality of the results.

In terms of turnaround time, very very effective - under 1 hour you get your result... which has been helping us to prevent some loss to enrollment in some patients. So that's what I say

**Interviewer**

...in terms of number of tests done.

**Number 1**

In terms of number of tests done, I can say that it contributed up to 30% of presumptives, quarterly.

**Interviewer**

Okay. That's very good

So number 5

**Number 5**

Okay, if I may say, it has been an awesome experience. As such it's highly recommendable. Since the result comes out on time compared to other methods of testing TB, so it's very awesome.

**Question**

Okay. What about the quality of results?

**Number 5**

The quality of result is fine, and it is faster, since it does not take much time.

**Question**

Okay. Then what about the turnaround time?

**Number 5**

The turnaround is very okay, because the Truenat machine anyway, like I said, it picks two samples at once compared with Gene Xpert that takes four and above. Then only that the Truenat cannot take presumptive of stool sample, but the Gene Xpert can do so.

If I may recommend it, it's very okay.

**Interviewer**

It's alright. Okay.

Number 3, if your network is not good, we will sort out how you can submit your responses; but if it is okay maybe you can respond to this number 4 question.

**Number 3**

My experience is that Truenat machine...

(Poor network)

Truenat machine relieves us from running here and there, getting to the lab or thinking where to take our samples to. Because the Truenat machine is in our local government, it has helped us a lot.

In terms of turnaround time, no time wasting. When I carry our samples there, within an hour or the next day we can collect our results.

Then it increase our case finding.

Then in terms of the quality of result, Truenat machine is very very good and it works faster.

Then in terms of MTB detection and even RIF resistance. So we don't have any problem with it.

Then when we receive the result, we place the patient on treatment immediately. So this makes our case finding to increase. We don't have any problem with it. Even when result is out, even lab technicians at the lab call us that the result is out. So when we have positive, they will call us to come and collect our result in time that is very good.

**Interviewer**

Okay. Thank you.

So number 5 question, I don't know whether it pertains to anybody. If I read it, if it pertains to you, then you just respond.

**Question 5**

**Can you share your experience with using the Truenat machine in the field, in combination with the PDX card to support testing for community acute case finding? What went well? What did not go well?**

Has anybody used Truenat for community acute case finding?

**All**

No

**Interviewer**

It's alright. So we skip question 5.

**Question 6**

**Can you tell us one thing you consider a major advantage and another you consider a major disadvantage with the use of the Truenat machine for TB testing?**

Number 4 I'm starting with you.

**Number 4**

The advantage of Truenat machine is that it's very reliable, it's cheaper, it's faster. Like the Gene Xpert, Truenat detect the DNA of a sputum sample.

Then the disadvantage of the Truenat is that any sample that is not of quality, it rejects it, or that have stains, It will also reject. Then it cannot take as many samples as the Gene Xpert at once. Also, the Truenat machine will reject any sample that is small, while the Gene Xpert can perform the test with the small sample. And to run the Riff resistant, after the MTB result, Truenat will require another one hour to run for RIF resistance. But Truenat has advantages like that, and it's cheaper, it's reliable, it's faster. The turnaround time is okay.

**Interviewer**

Alright. Thank you.

Number 5

**Number 5**

One of the major advantage of Truenat machine is that it is fast in producing result, and reliable.

One of the disadvantage, if I may say, is that it works only with two sample at a time. That is the disadvantage I think I have.

**Interviewer**

Okay.

So number 2

**Number 2**

One of the advantages is that it is...

(Poor network)

...very very costly. I can google the amount of each Truenat.

**Interviewer**

I didn't get you

**Number 2**

I'm offline then...

Disadvantage, when it comes to the time of engineer that have come to do the installation or whenever there's any mechanical fault. So the other one is the cost-implication of the machine itself - very expensive.

**Question**

When compared to Gene Xpert or...?

**Number 2**

Compared to Gene Xpert... In terms of turnaround time, it's very very easy compared to Gene Xpert. So that's what I can say about the advantage.

**Interviewer**

Okay.

So number 1

**Number 1**

One of the major advantages, apart from what they have said, is that in my area is light - ability to use it with the battery, it can still run. Because light has been a major issue in my area. So without that light we still can run Truenat machine since it as a battery. That's one of the major advantages.

Then the major disadvantage for us is that it does only two sample at a time. That is one of the major disadvantages that we have. But apart from that, it is reliable, it is accurate. I think the advantages outweighs the disadvantages for me

**Interviewer**

Okay. Thank you very much for that submission.

Number 3

**Number 3**

Advantages, Truenat machine is very reliable and acceptable. In terms of turnaround, Truenat machine doesn't waste our time. So it's working...

Then the only disadvantage that I can say is that it can run two samples at a time, unlike Gene Xpert where the modules are more in number.

**Interviewer**

Okay.

So I'm going to the next question. I'm starting with number 2.

**Question 7**

**Can you share your perspective on how the Truenat machine has affected the diagnostic journey/experience of patients undergoing TB testing services in your LGA?**

**Number 2**

Yes, I can share my experience because it's very very reliable for all the diagnosis of the presumptives. I can 100% share the experience.

**Question**

Okay. So explain how it has helped the experience of patients?

**Number 2**

Because it has contributed to our case findings. There increase in the case finding. Provided that there's quick diagnosis, the patient diagnosed can be easily placed on treatment so that there won't be spreading of infections... Instant initiation. Then there are some patients that are being diagnosed instantly, these patients will not be lost to follow up during the enrollment. You can easily place the patient on treatment. So... And I can share it that it has been approved by WHO in 2019, and it has been fully integrated into TB diagnosis so that others from LGA can be making use of it.

**Interviewer**

Thank you very much for that submission.

Number 5

**Number 5**

So far, it has been a good and welcome experience in my LGA in the sense that since the Truenat machine result comes out very fast. The lab attendant will now call; we will now call our patient immediately; we will place our patient on treatment, and do follow-up immediately. It helps. So with it we now say that Truenat machine has helped me a lot. It has been a good experience in my LGA. Because we use the Truenat machine, and that is why we generate the many cases that we submit every quarter. It has been a very good experience, compared to when we are using Gene Xpert.

Okay. Thank you.

Number 3

**Number 3**

In our facility, my experience is that...

(Poor network)

**Interviewer**

Number 3, see what we're going to do. Just remain with us, you respond to these questions and submit to me, I'll add it up as part of the interview. Because I'm seeing that the network has not been friendly where you are. Do you understand? So what two of you will do is to respond to those questions and forward back to me. I'll include it as part of the interview. So from our records you participated. Do you understand?

So we can move forward.

Now number 4, I'm starting the eighth question with you.

**Question 8**

What are your thoughts about the reliability of the Truenat test result compared to other diagnostic test?

Number 4

**Number 4**

My thoughts concerning Truenat is that it's been wonderful, it's been okay. It's reliable, it's faster, and it's even cheaper because they can even carry it. For us to use the Truenat, you don't need electricity... If there's power failure, if there's anything, the battery can be used to power the Truenat, and which makes the case finding easier. What we do in my facility, so as to reduce the time to initiate treatment, immediately a Truenat test is positive, a signal is sent so that they can call the patient immediately and the patient will be enrolled for treatment. So it's a good development, and it's good bringing Truenat to my LGA, to support the Gene Xpert. It's working fine and it has been helpful to us in my LGA.

**Interviewer**

Okay.

So number 4, do you mean that any test result from Truenat you consider it as being accurate?

**Number 4**

Yes, it's accurate

**Interviewer**

Okay

Number 1

**Number 1**

The reliability has been topnotch because we have been able to cross check the result from Truenat with that from GeneXpert and the result has been okay. The result has been okay, that is the truth

**Interviewer**

Okay.

So you can vouch for the accuracy of the result

**Number 1**

Yes...

(Background noise)

**Interviewer**

Okay.

Number 2

**Number 2**

Talking about the reliability of the results, I cannot say it's 100% effective. As we all know that Truenat and GeneXpert, they are just initial diagnostic tools. You cannot compare it with LBA culture and others because a times the Truenat gives error results.

I can only say it's 99% effective with the results because there are some others which I cannot compare Truenat with. One cannot compare Truenat with GeneXpert in terms of its advantages. You cannot compare the advantages... compare it with the PF ice, you cannot compare Truenat with FBA culture and other diagnostic tools. I can only say the result is very reliable, but one should bear in mind that it should be initial diagnosis tool

**Interviewer**

Okay. Thank you.

Number 5

**Number 5**

The reliability has a greater percent, let's say like 95%-97% reliability.

Okay

**Number 5**

Like I said, since we started using Truenat machine, at least it has been helping our case finding. And the facility where the Truenat are being sent to is where we generate more presumptive and positive cases, whether MTB or RIF-resistant. So it's reliable

**Interviewer**

Okay.

Let me start number 9 question with you.

**Question 9**

**Based on your interactions with other health care workers, which may include DOT focal persons, clinicians, nurses (these individuals who provide TB...), how confident are they with the results generated by the Truenat machine? Do they think that the test results are reliable?**

**Number 5**

Yes, they do because they know that the lab attendants have been trained for it. So once the result that comes out from Truenat, they believe it's reliable, because it's a qualified person that does the lab test and the person has been trained for it. So they believe it's reliable.

Okay. Thank you.

Number 2

**Number 2**

Yes, they do consider Truenat very very reliable because it has been approved by WHO and also it is in the national guideline. Since it is approved it means one should be making use of it. Then they all know that we are their supervisors and anything we say in the area of TB they accept it and consider it reliable. .

**Interviewer**

Thank you very much.

Number 4

**Number 4**

Yes, they consider Truenat results as being reliable, and they know that most of the health workers have been trained, especially the lab scientists that are doing the test. Also, during the initiation of Truenat in my LGA, most of the other health workers were invited, the laboratory scientists were also invited, and the information concerning Truenat was passed to all. So, most of the other health workers know about Truenat already. They know it is reliable.

**Interviewer**

Thank you very much.

So number 4, let me start the 10th question... We're almost coming to an end.

**Question 10**

**Can you think of any barriers to the scale up of the use of Truenat test for TB diagnosis to other local government areas?**

**Number 7**

The only barrier is that Truenat machine spoils easily and this makes work very difficult.

The factors that could affect carrying out the Truenat tests include lack of training for the laboratory staff, if the materials for the working of the Truenat are not available and if the Truenat machine is not functioning.

**Number 4**

What?

**Question**

What it's saying is that: you know now it's not all local governments that Truenat machines are available. Now assuming that there's need for a scale up to almost all the other LGAs, what do you think could be the hindrance to that scale up?

**Number 4**

Okay. What can hinder it may be the monetary issue.

Money? How?

**Number 4**

Where to keep the machine is of importance and could prevent the Truenat machine from being placed in some facilities.

Okay. Thank you.

Any other thing?

**Number 4**

No other thing again.

Health workers especially the Laboratory staff, those that will be handling the Truenat machine should be employed. They should also be trained, also they should also be ready to pay stipends to the laboratory staff in addition to their salaries.

Okay.

Could there be any other thing?

**Number 4**

Then accessibility again.

Okay. Accessibility to who now?

**Number 4**

To where the machine will be.

Okay. Okay.

Any other thing?

Do you think there could be anything in that facility that could make it possible not to carry out the Truenat test?

**Number 4**

It depends on the condition... Sometimes if there is no space in the facility for the lab where they're going to put the machine that could be a barrier because they have to create a structure before putting the machine there. Then, they have to negotiate with the management of the health facility. There should be a negotiation before putting the Truenat, because that one could also be a barrier. Then there must be someone, a Laboratory staff who is capable of conducting the test. If there no one to do that, it could also be a barrier.

Okay. Thank you.

Number 1

**Number 1**

I think one of the factors we can look at is personnel mobilization, as in those resource persons... Because we're talking about...

When they came to scale up our site and the local government, they took about two weeks or one month before they were able to. So the distance between one local government to another can also be a barrier too.

Then I think, just like the other person said, the persons available also... If the local government has qualified persons there to be able to handle the job. So I think those should be the barriers.

Okay.

Number 2

**Number 2**

I don't think there should be any barrier to that, provided that there's no misconception from other LGA about the use and reliability of this Truenat. Number 1 factor might be misconceptions about Truenat. If the things about Truenat are well sold and explained fully to the neighbouring LGA, I don't think there's any barrier of accepting Truenat machine for their diagnostic tools. Then secondly, depending on the personnel - if there's no personnel that can handle the machine, it might be a hindrance to it. That's two things I can say about it - misconceptions about this Truenat and personnel to handle it.

Thank you very much.

Number 5

**Number 5**

The only barrier that can occur if the LGA is not having a secondary health facility, like if the LGA has only primary health care where there's no lab. Like some primary health care; mostly primary health care, they don't have labs... the Truenat machine and install in primary health care where there's no lab technician and no lab there. It can now be barrier. Then the distance where the Truenat machine will be fixed; like the distance between the LGA and the neighbouring LGA where the Truenat is being fixed. It can cause a barrier. And this not producing multiple samples at a time is also a barrier.

Okay.

So let me reverse. Number 5, I'll start with you at once. I think this is the last question.

**Question 11**

**Can you think of factors that could facilitate the scale up of the use of Truenat to other facilities?**

**Number 7**

Before the Truenat machine should be taken to a new LGA, there is need for a meeting with the people of that LGA like the Chairman of the LGA, Chiefs, Medical Officer of Health and the entire health workers so that all will be aware of the existence of the machine in the LGA and how it works. Once this is done there will be no problem with the use of Truenat in that LGA.

**Number 5**

Okay. Truenat machine should be built in such a way that it will accommodate more than two samples at a time to enhance efficiency of the work.

Okay

**Number 5**

...to enhance the machine. So the factor I may say is that it should be built in such a way so that it can accommodate more than two samples at a time... So that it can enhance the reliability and fastness of the machine.

Okay

**Number 5**

So that's one of the factors, I think.

Okay.

Number 2

**Number 2**

Okay, thank you. What can facilitate it is all what we're saying. If one considering the advantages of this Truenat machine, one will accept it. Number 2, what can seem as if... It can be evaluated from the reports submitted... Provided they're generating a huge number of presumptives, it will take a lot of time for the results to be out. Other donors can make a look into it through their report. They might be saying that, through that their recommendation, there's need for Truenat in this LGA. That one can facilitate it, from their report generated. So that's the two things I think can facilitate the scale up - the good news about Truenat, and the early data or weekly or monthly or quarterly data reported to the national.

Okay.

Number 1

**Number 1**

I think one basic thing that stands out is training. If more people are being trained concerning Truenat, I think it will enhance the number of people that are able to use it very well, efficiently to produce better results. And also, if there's multiplication of the machine itself; it's not that in one local government you have one; but if you have multiple sites where you have Truenat, it will also help the scale up of the use of the Truenat.

Okay.

Number 4

**Number 4**

Okay. Number 1 is that not all LGA know about the Truenat machine. It's only some LGA that know here, although all the TBLs know and those that are working as a team know. Number 1; if there should be awareness, this can enhance the scale up of Truenat. And another thing that can scale up is: this Truenat produces two samples at a time; if this Truenat machine can test up up to four, four produced in a time, and it's being taken, it will reduce the workload in each LGA. Another thing is that to reduce the workload, it can scale up to four instead of two, which will make it more effective like the Gene Xpert to other LGA.

Okay. Thank you that submission.

I think that is our last question officially. I don't know if any of us has anything to say that you think was not part of the questions that were asked? Any comments, including how to improve the Truenat or the efficiency or whatever we see in Truenat today? Does anybody have any comment to make?

**Number 4**

Concerning the Truenat, instead of two producing, it should be increased to four, if that should be done. Then concerning all the, most of the LGA... At least in my state now, at least we have only in one LGA or two. If it can be scale up to three or four, so that it will reduce the workload of the Gene Xpert and the result will be faster - it will reduce the pre-enrollment gap, so that the patients that are being diagnosed immediately will be placed on treatment in time.

Okay. Any other comment?

**Number 2**

The effective use of this Truenat machine by all our lab personnel can at least keep a good record of all the work done, because if you're doing the tests without any proper recording of all the presumptives and results no one can assess the performance of the Truenat machines. But the effective use and good maintenance of this machine can encourage other partners to step in and supply more Truenat machines for those centers that do not have them now. So this will depend on how well we use, record and report all activities related to Truenat.

Okay. Thank you.

**Number 1**

This is just my thought. When they came, when the Truenat came, it was just an initial setup. I think with the advantages we have recorded, I think that should have prompted them to have increased the availability of Truenat in all corners of the country. Based on the positives we have seen, because like in the state I am, it's just the initial ones that were set up that is still there; there's nothing like an additional thing. So I think with the positives that we have heard, there's a need for a scale up the use of Truenat to other health facilities in the country.

Okay

**Number 5**

If it can be possible for the Truenat machine to be checking presumptives using stool sample, it will help us more... Because where the Truenat machine is being fixed, we have more of Paediatrics age group there and it is affecting our case finding adversely. This is because the stool sample lasts for about three hours. So when we have stool sample which we cannot use for Truenat, before the close of work, when the linkage coordinator will come and take the stool sample down to the other LGA where there is GeneXpert, maybe that will be more than four hours and the stool sample will be useless. So if there's any means that they can still make the Truenat machine to work using stool sample that will be good.

Thank you very much on that.

**Number 3**

My own contribution is that if Truenat machine can be taking more than two samples, maybe four, it can increase our case findings in our LGA. That is my own contribution.

Okay.

Thank you everybody for this wonderful participation we've had this evening. I appreciate it and I thank you very much for that.

**All**

Thank you, sir

Thank you.

Bye

**TRANSCRIPT OF KII ONE**

Q1 What is your impression about the TB burden in Nigeria and the effectiveness of the diagnostic network for finding missing TB cases?

Question number one is talking about my impression about TB being a burden. Nigeria is number six in the world with regards to the burden of TB and with less than fifty percent coverage in terms of regular molecular diagnostic platform, a whole lot of cases are being missed year in year out. A lot of people that we’re supposed to determine their rifampicin resistance because we use microscopy to diagnose them, we are unable to. So the Truenat intervention has helped us to be able to increase the number of cases that we are finding as well as increase access to a molecular diagnostic platform that can determine rifampicin resistance and help us place patients on appropriate regimen.

Q1B. the effectiveness of the diagnostic network for finding missing TB cases?

**Q** - effectiveness of a diagnostic network. That’s number 1, the B question.

**A** - The diagnostic network is so efficient. We spread the Truenat machines across different locations to make sure that they are being reached and in areas lacking molecular diagnostic platforms. The machines were actually placed in peripheral labs, where we felt these machines would function effectively. So I can say that we were strategic in the distribution process of Truenat machines.

Q2: What is your perspective about the use of the Truenat Tests for TB diagnosis and the location of the machines in the diagnostic network?

**A** - Yes. I said that Truenat diagnostic approach has been a game changer in finding all the missing TB cases especially in the outreach areas and communities because of its ability to work on battery. It is a battery powered equipment and it doesn’t require a whole lot of infrastructural upgrades before you can start using it. So, it makes it easy for us to get to those hard-to-reach areas and actually test, and you would see a location that is not having any case notification or one or two case notifications before could start notifying up to five cases or ten because of the presence of Truenat. It has actually helped in those areas and locations.

**Q** 3 - From your experience, what are the implications of implementing the Truenat Tests for TB case finding in terms **of Implementation Challenges**, associated operational costs, **efficiency of the intervention** and return on investment?

**A** - Yeah. I said in terms of challenges for TB case finding. The major challenge that we faced in the implementation of Truenat is staff attrition as I said earlier and that we have to continue to do supervision with training and re-training of the facility staff. Most times after training facility staff on the use of Truenat, the next information you will receive is that the staff has left the country for greener pastures. Then there was the need for power source to charge the equipment in some locations, and we were able to actually work around that by the provision of generator sets for some facilities, supporting some by giving fuel support to some that have generators while we provided power packs and solar panels for others. So we have been able to use that to reduce the impact of unavailability of electricity. Another challenge that we faced is the continual breakdown of TruePrep and that we had to contact a local agent for the fixing of the equipment anytime it spoils. So those are the challenges that we faced during the implementation of this Truenat for TB case finding.

**Q** - OK. We will focus now on the B component - associated operational cost.

**A** - Yes. On the associated operational cost so far, because it does not require electricity for its use, it does not require cooling system that you have to power the air-conditioner. So it was just minimal upgrade of the existing labs that we did and we were able to start using the Truenat for TB diagnosis. So in terms of associated cost, the cost is actually minimal, apart from the cost of the reagents and consumables, I think it does not require a lot of power optimization and the likes unlike what we see on other platforms that we have experienced in the past.

**Q** - OK. The next one is efficiency of the intervention.

**A** - Yes. The intervention is actually very efficient as we have seen that most of our facilities despite the challenges that they faced, they were not actually having a whole lot of issues around meeting their targets on weekly basis. So the intervention has been efficient and so many people have benefited from it in terms of TB case finding and Diagnosis across the implementation in our states.

**Q** - OK. The last one is the Return of Investment.

**A** - Although, just like I had said earlier, there is a great return on investment because with the equipment, we were able to reach out to many people and now we heard of the good news that Stop TB partnership and the Moldibo (referring to the manufacturers of Truenat) have been able to strike a deal that each test should cost around $7.00. Unlike what we have or what we have known for other alternative platforms, this is a plus in terms of cost of the test and the return of investment is actually good and the need to move the samples from a far distance to wherever the GeneXpert is available has been reduced and this is another advantage that has helped to reduce the cost implementation.

Q4 In your opinion, what factors act as enablers to a successful implementation of the Truenat Tests for TB diagnosis in Nigeria?

**A** - So I said that stakeholders’ engagement is what served as enabler and the engagement was all encompassing. It was from top to bottom, from NTBLCP to the community people to USAID and the Implementing Partners and all were ready to embrace the system. This has actually helped in the successful implementation of the project. We also ensured that all that were involved in the use of Truenat were trained. This massive training approach to all Lab staff helped in the successful implementation of Truenat.

Q4B Follow-up prompt – What would you consider barriers that would need to be addressed?

So the barriers that are we are able to address are ….we spoke about the breakdown of the TruePrep. This was a major barrier and there was also the need for us to use electricity to charge the system.

Another barrier is high attrition rate of the health workers, this time the Lab staff. We had situations where somebody is trained today and a few days or weeks later, you hear that the person has left the country and you have to go there and re-engage another person and start training all over again. So the high attrition rate is actually a serious problem because the testing platform requires a lot of hands-on, so anybody that is going to take over must have to be trained. The person would also require a whole lot of mentoring and supervision before the person can actually be able to handle the testing platform.

**Q5** - What operational advantages/dis-advantages have you observed with the Truenat Tests over the existing GeneXpert and TB LMAP in use in the country?

**A** - All what Truenat needs is actually a bench where to work and a place where to charge when necessary. It is battery powered and it can actually test for MTB and rifampicin resistance just as GeneXpert. But the beauty of it is that you can take it to anywhere even a remote area and you can start also use the machine, even on the field. You could use it as a point of care machine ie you can use it on the field and you can use as a near point of care ie you can use the machine in a peripheral facility that does not even have AC or electricity supply. Unlike GeneXpert that requires cooling system and if the environment is not cool, the machine would not work properly or not work at all and if there is no light, all the tests would be aborted, Truenat is not like that as even if there is no light, you can still work with the battery for up to 8 hours.

On the other part which is the TB-LAMP, TRUENAT has an advantage over TB Lamp as it has the ability to detect rifampicin resistance. You have to know the rifampicin resistance of the patient so you will be able to place such a patient on appropriate regimen. For TB LAMP, if the test is positive, you have to subject that test to another test to confirm the resistance before that patient could be placed on treatment. But with Truenat, as soon as you are done with the testing, you would know how to classify the patient and then you can place the patient on the appropriate regimen. So that is the advantage that Truenat has over the TB-LAMP.

**Q** - Thank you sir, now we go to the B component. So what operational disadvantages have you observed with the Truenat test when compared to the GeneXpert or TB-LAMP?

**A** - What I have observed is the fact that Truenat requires a whole lot of hands on, manipulation, when compared to GeneXpert. The skill of the person handling the Truenat has to be top notch in terms of the pipetting and doing the appropriate things because there is a whole lot of hands-on. Unlike GeneXpert that you just have minimal hands-on, you load your cartridges and you are there. So that is the difference between the GeneXpert and Truenat, on just the technical part of it. But what we have experienced so far with the Lab staff on the use of Truenat is that after some time, the laboratory personnel get used to it and they adapt to the system. But that as it may, is actually a kind of advantage which the GeneXpert has over the Truenat. Also, unlike Truenat where rifampicin testing is a reflex test – a test you have to do after the Truenat has turned positive, ie MTB positive and you now have to go ahead and do another test with the same machine to determine the rifampicin resistance, but from one straight test, the GeneXpert would diagnose a patient as having TB and also determine the rifampicin resistance status of such patient. So that is another advantage that GeneXpert has over Truenat. However, it depends on the perspective that you want to look at it from, because many of us now view this aspect of Truenat as an advantage rather than a disadvantage. This is because you will be able to process more samples when you test and it is negative. It means that test is completed and you move tp another test. Rather than the test where you would subject the testing to the 2 tests together you just test quickly and then move on as long as it is negative. So it is only positive that you would have to go further. That not-withstanding, the fact that you have to do further manipulation unlike the one where everything happens in the machine, is a disadvantage.

For the TB-LAMP, the advantage it has over the Truenat is the fact that the TB-LAMP has a very high throughput that is at once, the TB-LAMP can run 16 tests – one positive and one negative and 14 patients’ samples at once within 2 hours. So by the time you are using almost 2 hours to run through Truenat which is two tests with the Truenat machine we have now, you will realize that the 2 hours, you would use it to run 14 samples using the TB-LAMP. So, the TB-LAMP has a very high throughput in terms of the number of tests it can run at once, that is the advantage.

**Q6** - So we go to the sixth question sir. What are your views about the use of Truenat for community based acute case finding activities in combination with ultra-portable digital x-ray services?

**A** - Yes. So far, we have used the machine in a portable form, that is on the field as a point of care three times, and we had similar experience the first 2 times that we used the machine in that form which is the time that it takes for you to pack the machine from the site, to unpack it when you get to the field and the time it takes for you to run the sample on the field. You cannot run much samples and at the end of the outreach, you still have to pack the machine back into the box. When you get to the facility, you unpack. So we see it that with the current situation that we have, which is the fact that we have a whole lot of samples that need to be tested using the regular platform, and its not as if we have a hundred percent saturation of molecular diagnostic platform yet, so it would be better for you to allow the machine to the near point of care rather than taking the machine to the community to use. But of recent, we derived another method which actually, we had a better experience in order to increase the duration the machine would work on the field. We did the packing of the machine a day before the outreach and transported the machine to the outreach area a day before, so on the next day, before the outreach even commenced, the machine is already set up. So as patient samples are being collected, they are being processed immediately. And we were able to process more. So, in that view, we believe that if we can combine the machine and a particular portable digital x-ray for community activity as a permanent machine to that, or increase the number of modules - now we are using a 2 – module machine to support the work. If we can get a quadro and permanently attach the quadro to a portable digital x-ray system for community activity as in solely for community activity so you don’t need to be moving the machine between facility and the community and will now require you packing and unpacking and then quickly packing again, it will actually help in having a better output because the output in our last outreach is actually better than the previous ones that we had. So, in a nutshell, what I will suggest is that a bigger machine that can run like 4 samples at once so that it will be able to cover for the number of samples that is being generated on the field during the outreach and that is just attached permanently to the x-ray system, maybe a mobile truck or maybe the two of them carried together like we have in some cases that have happened in the past. So because we don’t have enough now, we cannot fix it for the acute case finding alone. We have to keep using the ones we have within the facility which will require packing, unpacking and re-packing again to the facility. So if the process of picking up and taking back to the facility has been removed by giving that machine to the truck or portable digital x-ray permanently, then we are good to go. It will reduce the number of time and if we have the bigger system, it would cater for more samples. So the combined system would be the best.

**Q** - OK. You’ve made a good classification or analogy in your comparison between the Truenat and the GeneXpert and TB-Lamp. Hence, this question becomes very important. If you are to advise the manufacturer, what and what would you tell them on how to make the Truenat test more efficient in TB service delivery,

Q7 What advice will you give to the manufacturer to make the Truenat Tests more efficient in TB service delivery?

**A** - The advice I would give the manufacturer is to improve their TruePrep – the technology of the TruePrep, we are not there yet because it breaks down easily. And secondly to look into production of a higher module machine that can actually test more. So with that, if you have like a machine that has up to 20 modules or 21 modules or 30 modules, you will be able to cover a whole lot of ground since it doesn’t require a lot of infrastructural requirements.

**Q** - Do you mean 21 modules at a time?

**A** - We have 1 module, we have 2 modules, we have 4 modules. If they can increase the number of modules and improve the technology of their True Prep so that it would not be breaking down easily, it can go a long way. I think they are working on production of battery pack and solar panel to support the machine for continuous charging so that the power would not be a challenge anymore. It is also another thing they should buckle up and release it to the market as soon as possible.

**Q** - OK. Any other advice?

**A** - Any other advice I can give to them is the way the thing is now, we should look for a way of scaling up the number of platforms in the country so that most of the challenges we are facing now would be addressed and people can be reached and start using the platform to test without any interruption and we would be able to find more cases because as we are thinking of increasing the number of cases, we should be thinking of increasing the diagnostic platform also. And also look for support all around the world for us to get to at least up to 100 percent coverage in the country in terms of molecular diagnostic platform.

**Q** - OK. Just the last question. Is there anything else you would like to talk about the Truenat?

**A** - Nothing else, I’ve touched virtually everything that is peculiar to Truenat and things we’ve enjoyed so far and what we think they should improve and I believe if they are able to take care of that, we would be good to go.

**Q** - OK. I think that is just the only question we are missing. So is there any other comment you have to make, sir?

**A**. - I’d just want to say thank you.

**Q** - Thank you very much and thank you once again for your understanding of the situation. I appreciate so much.

**Title of Interview/discussion:**

**KEY INFORMANT INTERVIEW 2**

| **Interviewer/facilitator/questions** | **Respondent/responses** |
| --- | --- |
| What is your impression about TB burden in Nigeria and the effectiveness of the diagnostic network for finding missing TB cases? | Okay let me just describe the question- TB burden and the effectiveness of the existing diagnostic tools for finding missing TB cases; is that your question? |
| Yes ma | Okay, the TB burden in Nigeria is high and Nigeria is one of the high TB burden countries of the world, the first in Africa. The effectiveness of the tools we have: in Nigeria we actually use a lot of TB diagnostic tools- recommended diagnostic tools recognized by WHO. Most of them are quite effective. Actually, on the path of diagnostic tool usage, I will mention GeneXpert first and GeneXpert is quite sensitive and we adopted it in 2011. We have been using that until we had the order to complement what GeneXpert is doing because of the challenges we have encountered in this country in the utilization of GeneXpert. So currently we have an array of diagnostic tools. We have the GeneXpert as I earlier mentioned, we have the TB LAMP and we also have the most recent, the Truenat.  The conventional ones we have and we have been using are the cultures, LPA and the AF Band those ones have their limitations. So, I must say that we are rich in TB diagnostic tools in this country and we are privileged to have them and they have actually been very effective. The merit of one override the demerit of the other. No diagnostic tool is 100 % efficient; however, we actually marry all the diagnostic tools in the field to get optimal result in terms of our TB diagnosis in Nigeria. I think I have answered your question or are there addendum to that which I have not answered? |
| the only addendum to that is: do you think we have been effective in all these diagnostic networks in finding a missing case? Has it been working for us? | To a greater extent, the answer is Yes; we are doing our best but we still need to do more in order to optimize these tools. We are doing great but there is need for improvement. |
| What is your perspective about the use of Truenat tool for diagnosis and the location of the machines in the diagnostic network? So, we will start with the first one: your perspective about the use of tuners tool for TB diagnosis? | Okay so the Truenat is the most recent TB platform we added to the many of the diagnostic tools in the country. My perception of Truenat is positive, Truenat is actually a welcomed tool because of its ability to detect RIF resistance. That is a plus over other diagnostic tools that do not have this property. Truenat also is highly sensitive. From the experience in the field, we have been able to detect TB bacilli and TB yield is relatively high and you can also compare that with what we were getting from GeneXpert.  So, my perspective is that Truenat is actually a veritable tool for TB diagnosis in Nigeria. It also has helped us in expanding access to TB diagnosis in the country. The fact that it uses in-built batteries means that it can also be used in peripheral labs- that is low level labs where we have issues with low electricity. The battery helps us to test even in challenged sites and hard to reach areas with very difficult terrain, so it is a great tool for us and I have a positive perspective for the tool. |
| So, what about the location of the machines in the diagnostic network? How do you see the location of the machine? Is it favorable? Is it okay? | We have 38 Truenat machines in the country currently. We were supported in acquiring the machines, we got them from USAID. That is to say that USAID is supporting the TB programme in Nigeria. In the distribution, we have a fair distribution compared to other diagnostic tool distribution we had been using before because we learnt from our experiences with other diagnostic tools. We learnt from our mistakes and we were able to do our mapping. We have done our site selection and the installation of machines. So, for me, the distribution is such that we have a better coverage and good distribution. However, the coverage is still poor we are yet to have them in other States of the federation. A lot of States do not have Truenat We need to have them in all the States. Truenat is actually located in TB LON States; so, it will be a good thing if we have them expanded to other States so that they too will benefit from what we in TB LON States have been benefiting. Thank you. |
| Okay, now from your experience; what are the implication of implementing Truenat test for TB findings in the terms of implementation challenge? Okay, first implementation challenges; second is associated operational cost; third is the efficiency of the intervention and the return on investment. You may take them one by one. So, from your own experience, what are the implication of implementing the Truenat test for TB missing case findings in terms of implementation challenges. We start with that one. | For implementation challenges, we actually faced alot of challenges when we started the implementation of Truenat. One of such is that we had issues with the charging of the tools in as much as we have inbuilt batteries; there is also need to charge the Truenat platform. There was a misconception when we started. Before we started implementation, we felt that the presence of the inbuilt battery had actually addressed the power issues. So, we never knew that even though there are inbuilt batteries that there was need for us to charge the batteries. We felt that electricity will not be a challenge again as we have gotten them into the country and started training, that was when it dawned on us that we actually needed electricity and as such, we started battling with sites that do not have electricity supply because in most of these sites especially the low-level ones; there were no electricity at all and we never had alternative source of power. That was a huge gap for us. Because in these sites, they were either renting generators to charge the Truenat machines or they were taking the Truenat machines to another site to be charged before bringing them back. It was not until recently when we started working toward getting a solar system to support sites with power issues. That one is still on-going. We want to believe that before the end of this quarter those sites will be supported with solar system such that there will be no need for us to take the equipment outside the facility for charging. That is one. Again, we found out that those Truenat machines because their locations or the desired locations were to be at the peripheral labs where we have neither GeneXpert nor TB LAMP, those sites that are in hard-to-reach areas; we found out that we had Human Resource issues in some of the sites. So that was also a key consideration when we were introducing Truenat. So, for some of those sites, we supported with Human Resource and that was additional challenge on implementation. Another challenge we encountered was that for some sites, the errors were very high because Truenat is highly sensitive. It needed a lot of STEPS unlike GeneXpert on the part of the Lab staff.  So, the staff needed to be trained very well. If you don’t have a dedicated staff, it will be an issue. For most sites, they were perpetually recording high errors in the sites until we had follow-up supervisory visits to correct that. Lastly, the one I remember is: when we started, the utilization rate for Truenat was very low across board. That was because there was no sensitization meeting during the training on the use of Truenat. So, when we identified that, we quickly carried out sensitization meetings for key stakeholders in the States and we made them understand that there is a diagnostic tool in the State. And that was actually what prompted them to start sample referral and all that. Again, sample referral system was also an issue because we found out that for GeneXpert sites that could not take all their samples, we found out that there was need for us to re-distribute samples to Truenat sites and that was not forth coming with the agency in charge of sample referral; so, we had to engage the agency again in different dimensions before they could accommodate the Truenat sites in their network. So all those little challenges that we experienced though most of them have been overcome if not all. |
| Okay. I am sure you have almost covered all of them though I segmented it. We have talked about the implementational challenges as it related to operational cost, I know you have said one or two. Is there any further elaboration you want to put there? | Okay the HR cost |
| (cuts in) the associated operational cost | Okay, the operational cost was actually moderate because the Truenat platform and the reagents came as a gift, a donation from USAID.  The only operational cost that we encountered as Implementing Partners were the lab upgrades and the associated assessments we did for site selection and that were just minor things we did. So, it was a moderate cost. That is during the pre- implementation phase. |
| Okay. So what is your view on the efficiency of the intervention and again the return on investment? | Okay, so the intervention is quite efficient; the TB yield is quite high. Since it is a tool that has high sensitivity, I think the return on investment is very encouraging. The TB yield is higher than 10% which is higher than average TB yield. That is the desired TB yield in Nigeria TB programme. So, it is a veritable tool for us. |
| Okay we move straight to the fourth question. | Okay |
| In your own opinion, what factors act as enablers to a successful implementation of Truenat test for a TB diagnosis in Nigeria? | What factors act as what; I didn’t get the question. |
| Act as enablers for a successful implementation of Truenat test for a TB diagnosis in Nigeria? | Okay, enabling factors to a successful implementation is: (1) Great support we received from the National TB programme. The collaboration we had with the National TB programme made implementation very seamless. The sites were carried along. So, to speak, a collective effort of everybody- the Implementing Partners, the funders, the State TB programme (everybody was operating on the same frequency) and that brought about seamless implementation. Even the authorized local service providers everybody were carried along and operated from the same frequency- that was a very good enabler for us. (2) Again, the fact that when Truenat came on board, we were having issues with GeneXpert and Truenat was less dependent on environmental temperature (that is air conditioner); it also enabled seamless implementation. Given that the cost implications were not so much, it also enabled seamless implementation of Truenat. Given that the cost implication in setting up the lab were not so much, so it also enabled seamless implementation of Truenat. Those were the few ones I can remember now. |
| So. we go to the B part of it: what will you consider barriers to the successful implementation of Truenat test diagnostic for TB testing in Nigeria and what could be done to address such barriers? | Do you mean barriers to |
| Barriers to successful implementation(cuts in) | what are the barriers? Okay, the barriers that has come to mind is connectivity. You know linking up Truenat to digital connectivity network. We are still not yet there. Though a lot has gone into that; but I believe that when we get there, implementation will improve much more. We are yet to get there and it is also a barrier to a successful implementation because it could have helped us in monitoring a lot of things like reagent monitoring, error rate and so on. I think if this is not done, we have to embark on monitoring visit to the sites to do that and that is when things will get better. So, it is a barrier for a successful implementation. You remember the point I made earlier about the fact that some sites do not have electricity and even alternative power supply. So, for those sites, you will see them not testing most of the time because the support for fuelling is not sustainable as it is difficult for such request to be honoured all the time. So, you see them going to another site to charge their equipment; so, it is actually a barrier to a successful implementation. In terms of human resource, when we started implementation, we actually experienced low utilization. A lot of sites were not fully utilizing the tool because the National TB programme then were still offering incentives to GeneXpert operators; so, incentives were provided for them and the incentive was performance based. That is to say that as you test, you will be motivated for testing more. That was not done for Truenat sites. So, you will see a lot of Truenat sites not happy about that- there was no zeal, no motivation for them to utilize the tool not until Implementing Partners also supported them with stipends. So, it was when we supported them with stipends that we saw the utilization rate going up. |
| So, we go to the fifth question: I think it is in two parts so we will start with the first part; what operational advantages do you think we have using Truenat testing over the existing tools like TB LAMP in the country. | Okay, operational advantages. I think i will first start with Truenat advantages over GeneXpert after which I will talk of that over TB LAMP. The advantages of the use of Truenat over GeneXpert is quite enormous. The first being the Truenat platform itself. Okay the first is the cost implication between GeneXpert is not much; the difference between the cost is not much. |
| Which one is higher? | 9-dollars, 8 dollars so it’s not much or significant. However, in terms of operational cost, getting the lab ready, maintaining the laboratory and sustaining testing; the cost of GeneXpert is much higher. We should think towards the direction of providing air conditioner, making the air conditioner work all the time, making sure you have the inverters on, and so on and so forth. The cost of GeneXpert is much higher than that of Truenat. Again, just like I said, no tool is 100% perfect, if it has a merit on this, it will also have a demerit on the other hand. The fact that Truenat machine has two modules, it had also limited us on the number of samples to be processed unlike GeneXpert. Again, GeneXpert can be used to test other samples other than sputum especially stool for childhood TB diagnosis and that is not obtainable for Truenat. So, there are merits and demerits. Again, one key one I don’t want to forget is that we can use Truenat in a mobile fashion, in community interventions. If you take Truenat to the site, it will enable you make diagnosis same day. And this is not possible with GeneXpert. It is a very big plus for us and that has actually helped us in our community interventions where we take Truenat; we coupled Truenat with PDS, take them to sites, we do screening and as we are screening, we are getting the Presumptives. So we do Truenat test at the spot and get diagnosis the same day and put the patient on treatment. It reduces Turn Around Time for patient management and also has reduced testing falling out in the diagnostic pathway. Thank you. |
| Okay, I think you focused so much on the GeneXpert. Can you compare the advantage of Truenat over TB LAMP? | Okay, Truenat is always preferable over TB LAMP for some reasons; first being that Truenat is able to test for Rifampicin resistance; that TB LAMP cannot. Although this has not been validated in the country but the report, we have has it that Truenat is a little bit higher in terms of sensitivity than TB LAMP. So that is also a plus. The fact that Truenat has an inbuilt battery and TB LAMP has a portable solar system; there is no merit or demerit on that. The merit of Truenat over TB LAMP is the fact that you can use Truenat to test for Rifampicin resistance |
| Okay, you have almost compiled all of them so, I am now coming to the disadvantages of Truenat with GeneXpert and also with TB lab? | Do you mean the disadvantages? |
| Yes, the disadvantages of Truenat when compared to GeneXpert and also when compared to TB LAMP. I know you mentioned them but I may need for you to state them again so it might help us. | So, the disadvantages of Truenat over GeneXpert: (1) just like I mentioned before that Truenat are limited to two module machine meaning that we have limited number of samples to test; but for GeneXpert, we have moved on to more than four module machines. Meaning that we can test two times or eight times of what Truenat can test using GeneXpert. That is a disadvantage in terms of Truenat usage. Another disadvantage is that you cannot use Truenat for stool and other specimens. It is strictly forsputum for now while for GeneXpert you can actually use it for extra-pulmonary TB diagnosis using other specimen like stool So those are the main disadvantages. |
| So,can we extend it to that of TB LAMP now? I know you have mentioned them before but there is no harm in hearing them so many times. | No problem, the fact that Truenat is limited in use and can process 8 -10 samples in a day while TB LAMP can process up to 70 samples in a day. So, the use of Truenat for community intervention when we have large number of samples to test is actually limited. We limit what we can do when we are using Truenat compared to when we are using TB LAMP. Another disadvantage over TB LAMP is that TB LAMP is kind of durable and stronger. And can withstand frequent transportation- that is road transport during community intervention while Truenat is a little bit fragile compared with TB LAMP. We record more wears and tears when we use Truenat compared to when we use TB LAMP. Again, Truenat does not come with solar panel package while TB LAMP has that package and it is actually helping us in hard-to-reach areas where we don’t have electricity. And we have also used that in outdoor testing unlike Truenat that we need to charge before going to the field. With TB LAMP, all we need do is to identify a sunny spot and you are good to go with your testing. So those are the disadvantages over TB LAMP. |
| Thank you very much. So I go to the sixth question. What are your views about using Truenat for community -based acute case finding activities in combination with other portable diagnostic system? | I have earlier mentioned that but I can always repeat them again. So Truenat can be used in a mobile fashion unlike GeneXpert. We have actually used that on what we call ‘Wellness on Keke’. Wellness on Keke is a new innovation by KNCV where we deployed the tricycle (ie the keke); we also deployed the PDX(ie the Portable Digital X-ray) and we coupled that with a diagnostic tool either Truenat or with TB LAMP and we take them to the field for TB diagnosis. This has shown to be very effective because the digital X-ray is very sensitive, it uses artificial intelligence and from it we were able to get true presumptives.  Also, we were also able to profile them for TB diagnosis using the Truenat and while we are going to the field, we go with TBLS and also community mobilizers. The TBLS put those positive on treatment right in the field hence no one loses a patient; everything is done like a one-stop shop for us. So that is basically the advantage of the use of Truenat when coupled with PDX. |
| Thank you. I think this question is very important. You have done a very good description of the advantages and the disadvantages of Truenat over GeneXpert. Now if you are to advise the manufacturers of Truenat machines, what and what will you tell them in a way to make a Truenat test more efficient in TB service delivery? | Okay. The first thing which we have actually talked about is for them to bring in solar system like the one we have for TB LAMP since they are targeted for lower level laboratories in the communities that may not have power support. It is very important and very critical that it comes with solar system so that it can be used anywhere anytime and we will not have issue of power. I will also advise them that they should validate Truenat using other specimen because the way to go now is not the use of Sputum for TB diagnosis; it is the use of other specimen (especially for children) that are user-friendly and are easy to get like stool. If we are able to validate this using Truenat and the results are okay, I think it will go a long way in solving a lot of issues we are having in TB diagnosis especially in the hard to reach areas where we have installed Truenat. so basically, those are the two things I will advise them to do- to validate using other specimen and also to get solar system to support the platform. |
| You will not bother about the modules particularly when compared with the GeneXpert; are you comfortable with that? | They have quodro machine just that Nigeria have not adopted that. |
| Okay. | Manufacturing, it is there ; just for us to change like the GeneXpert You know initially we started with GeneXpert with four modules before we found out that we can actually expand to sixteen modules. It was there in the market. So we just went to buy four modules for GeneXpert machine. |
| That is good, thank you for that. So is there any other thing we advise them apart from validation and the use of other specimen? | Those are the ones that comes to mind. Okay, again for the inbuilt batteries, if it can be removed and charged somewhere else. That is to say if it will be removable from the equipment. You remove the battery and go somewhere else to get it fully charged. It will be very good for operators. Rather than fixing it to the machine and it will not be removed and carrying the machine alongside when you want to charge it. So that is not really convenient. |
| Thank you very much on that; I am sure you must have exhausted all your views but this is the last question. However, the eight question is just for you to say something: is there anything else that you may want to talk about on this Truenat issue | There is nothing to talk about other than that the local service providers should get more of the spare parts because initially we were not having enough machines breaking down but now it is taking longer time for them to resolve issues as a lot of machines pack up. So if we are able to get spare parts readily available and expand the pool of machines we have while having in mind that very soon we will have more machines as high as 300 machines in this country. We just have to brace up with that upcoming challenge as promptly as possible |
| Thank you so much; I am so delighted having this interview with you | You are welcome. |

**KII 3**

| **Interviewer/facilitator/questions** | **Respondent/responses** |
| --- | --- |
| I will go straight to the first question Sir. What is your impression about the TB burden in Nigeria, and the effectiveness of the diagnostic network for finding missing TB cases? | As you are aware, Nigeria is in position six globally in terms of TB burden and in Africa, we are in position one, and if you look back and look at our previous notifications, during pandemic in 2020, Nigeria stood out, we notified over 38,000 cases. In 2021, we also made a break through which was celebrated all over the world, just beyond the shores of Nigeria, we Nigeria also notified over 207, 000 TB cases. Last year in 2022, we have also followed up on the progress report, notifying over 285,000 cases. So, those cases are here, it is huge, the more we contribute and support the system, the more cases we notify. |
| Okay. So, we go to the effectiveness of diagnostic network for finding missing TB cases, how effective is it? | Yea. If you look at these records I gave you earlier on about notification, you can also trace it back to the support we are receiving in terms of adopting the WHO rapid diagnostic tools. Recently, we have seen the contributions from new tools like GX PAD that existed since 2011 and then the new entry which is the Truenat, MTB diagnosis and then the TB LAMP, so all these new tools have also contributed effectively to this notification support that I spoke earlier. |
| Okay. So, I will go to the second question Sir. What is your perspective about the use of the Truenat test for TB diagnosis and the location of the machines in the diagnostic network? | Yea. If we group the diagnostic tools which we have now, GeneXpert, Truenat and TB LAMP, we can say that the 3 are still giving us less than 51% local government coverage and if we speak specifically on the Truenat, following the global recommendation on Truenat, we adopted Truenat in 2011 with the approval by the Hon minister of health when we have followed the due process of adoption and after that, we had engagement with stakeholders and as you may be aware, the USAID through the KNCV and other partners like the IHVN supported the national program with 38 Truenat machines, they were all 2 modules, at the moment we have 39, there is also a private sector in Lagos that is supporting us. So, we will say that what we have now in terms of Truenat and specifically for the locations where they are now, is still low, we expect to get more of these tools to be able to run with the targets we have nationally and internationally. At the moment we have limited number of Truenat machines, we are hearing good news from our stakeholders saying that more of these tools will also come into the country in the next few months or years. That is the position |
| Okay, but how do you see the use of Truenat, has it lived up to expectation sir? | Yes, Truenat is designed to be placed at all levels of TB diagnostic network because it has minimal infrastructural requirement, it is also powered with battery and it is also what you can use in a room temperature. Truenat does not require us to use air conditioner, it does not necessarily require the use of other complicated equipment like biosafety cabinet. So, to a large extent, we will say yes, that it is meeting up with expectations that also means that it does not have limitations. |
| Thank you Sir. I will go to the third question. From your experience, what are the implications of implementing the Truenat test for TB case finding? In terms of (1) implementation challenges (2) associated operational cost (3) efficiency of intervention and (4) return on investment. I will start with number 1, from your experience Sir, what are the implications of implementing the Truenat test for TB case finding in terms of implementation challenges? | Yea. Implementation challenges, we still require electricity to charge the batteries and you know the power issues in developing nations like our own and then Truenat requires good level of skill for laboratory personnel for manipulations because there are different phases, you will be first of all be required to detect MTB and then proceed to obtain the RIF status. As you are aware, the battery for Truenat is not detachable, when we talk that we can use it to do this and that, we can easily move it around, we should also note that the battery is not detachable. Technically too, Truenat does not detect very low cases of MTB cases and the fact that we have different chips for MTB is also a limitation I believe with time we need to look at how we engage the stake holders or the product producers to also improve on that. |
| Okay, let us go in terms of associated operational cost. | Operational cost, except we do some deep down analysis to compare it with others, of course we know the cost of GeneXpert machines. For instance, we know that the 4 module machine is about 17 US Dollars and we also know that each cartridge for ultra is about 9.98 Dollars and then we talk of other cartridge we use at the level of integration. So, if we look at it and comparing it to what we have in Truenat, we can say that is lower, we also know that we are using other molecular tools which we may need to so some deep-dive analysis to actually come up to say yea, it is minimal. Over all, I think we also embrace it to say it to say that it is doing what we want it to do for us. |
| So, we go to efficiency of intervention and you may combine it also with return on investment. | Yea. It is all about engagement, we can straight away say that having embraced and having also engaged key stakeholders beyond the public sector and private sector, I think we can say that there is a great benefit in that regard of investment return. |
| Okay, we will go to the fourth question Sir. In your own opinion, what factors act as enablers to a successful implementation of Truenat test for TB diagnosis in Nigeria? | Yea. Straight away, we have stakeholders’ engagement. We have found that for every technology, every tool, once we start with engaging stakeholders, everyone including the private sector, then we will be able to drive it. The entry of Truenat was well organised because all have embraced what we believe in which is stakeholder engagement. We actually engaged them in a national program and we brought on-board every other key stakeholder. The process of adoption was part of what made our implementation easy and awesome because we engaged everyone from the point of developing the appropriate guidelines and tools and also capacity building, trainings at different levels. Those were enablers that created good environment for everyone to embrace this new technology and then we are able to run with it. |
| We will go to the reverse side of the question, so what would you consider as barriers that would need to address for a successful implementation of Truenat test for TB diagnosis in Nigeria Sir? | Yea. We are aware that Truenat machine is produced by another country and there are some groups of individuals that are doing that, who are those people, if you ask Nigerians, where is Truenat produced, many people will not be able to tell you but Truenat is produced in India, Moldibo team. So, they are not visible to us, government at all levels needs to also embrace these people, so that when we see them it will also help us in coordination, we need to see these people, we need to have a forum once in a while, we bring these people on-board, they will also help us in advocacy, we need to see the people that are producing instruments/equipment that are helping us to solve our problems. So, we need to look at engaging them and some other stakeholders that are pushing for us to adopt these technologies and continues to use it, that will actually help us. Also, as I pointed out earlier that there are some few technical things that needs to be looked at. The issue of detachable battery, issue of detecting MTB and then proceed to obtain the RIF status and some of those things. These are the things I believe will also improve our implementation of Truenat. |
| I go to the fifth question Sir. I think we are moving on a good note. Thank you Sir. What operational advantages/disadvantages have been observed with the Truenat test over the existing GeneXpert and TB LAMP use in the country? Maybe we start with the advantages that the Truenat test has over the GeneXpert and TB LAMP. | Like I mentioned earlier, these instruments do not require air conditioning, so, it is flexible. It is designed to be installed and placed at any level of our laboratory network, we have the peripheral level, the state, the zonal, the national, so at all level of our laboratory network, we can place Truenat, which is a very big advantage. It is also battery powered and uses room temperature, and the course reagents are also stable. All these are part of why we feel that Truenat has also come to support our system. |
| Alright. We will now look at our operational disadvantages that Truenat has when compared with GeneXpert and TB LAMP | We are looking at the fact that Truenat has 2 modules, what we have in the country now is 2 modules, the 38 machines we have are 2 module machines, which means, if you do active outreach program in some rural areas and you generate huge samples like 150, where will you store them to be able to analyse, I want us to look at the fact that we don’t have the 8 modules, although we are hearing there are 4 modules which we believe it will also come to the country. At the moment, we are using 2 modules, in places where we have placed them, how do we store our samples since we can only run 2 samples at a time. That is a very key operational challenge which you have also seen because it will make us to look at sample referral and again because of manual manipulations of sample and other reagents, you also need to settle down and train the technical team in the country. It needs a dedicated training to be sure that people can actually do this work and do it very well. So, these are some of the key things we believe that if we improve can help us to make more progress. |
| Okay. You have mentioned community based case finding, what are views about the use of Truenat for community based case finding activities in combination with ultra-potable digital systems? | Yea. Every laboratory diagnostic tool will work well in complementary with every ultra-potable digital X-ray, so we recommend and we appreciate, if we can have more of these because they will also help us. Decisions on findings is not only restricted to laboratory findings alone, clinical history and other meaning from clinician and other health workers will also help us to find improve what we are doing with respect to diagnosis, beyond just laboratory. So, we believe that they can complement each other in order to improve what we are doing. Thank you |
| Thank you very much Sir. So, you have a very good analysis of the Truenat, we are now seeing you as an adviser. So, what advice will you give to the manufacturers of Truenat to make it more efficient for TB service delivery in Nigeria? | Yea. The level of awareness of TB among health care workers and among general population is still low, it is less than 30% in Nigeria and so, we want to see how we can get support from manufacturers, product producers to also walk with us in a program to create more awareness because it is when people know what you have before they will be able to use it. Some of the clinicians do not know of the use of Truenat and they will not even make request for laboratory test. So, even if you have the machine and you don’t have awareness among appropriate persons that will make request for it. We need to do more on awareness creation, we need to do more on coordination, we have Truenat, we have TB LAMP and we have GeneXpert. Then you ask me, how many coordination forum is happening to let the people understand that this is what we have but it is not happening to the level we want it? So, we believe, manufacturers can support stakeholders in Nigeria, support National program to also do more, so we advise them to also do more on coordination forum, and advocacy also, there are different levels of government as you are aware, local government, state and then national. National assembly members, if you go to the senate or upper House, that is House of Reps and ask, even among the clinical team about these tools, they may not tell you anything about it, they may not even have heard of it. So, we need to step up out advocacy to this level so that government will bring up money to support our resource mobilization approach. Thank you |
| Okay. You mentioned something very important about engaging the manufacturers in forum whereby there will be a meeting between the manufacturers and the TB team in Nigeria. Now, if such a meeting will ever hold, is there anything you will ask them that might help them to at least improve efficiency of Truenat machine? | Yes. They will be aware of who is procuring the machine, they will also be aware of success stories from different countries, so we will ask them to share experiences of what they have from other countries. Like you may be aware, last year, I was also supported by some TB partnership and USAID to speak on Truenat in South Africa. So, if we bring the manufacturers, we will tell them to share experiences of what is happening in other countries in terms of success stories, in terms of challenges. Also, by hearing we will also adjust our system to say that, these are the challenges there, let us see how we will adjust our system and face the challenges there and how they were able to overcome it. So, such forum will provide opportunity for a good interaction, for us to learn from one another and improve on the service delivery. |
| If you are to talk one on one with the manufacturers and tell them areas to improve upon, as far as Truenat machine is concern. | So, if I am to speak with them directly, I will remind them as I have explained earlier, I will remind them that there are many manual steps in using Truenat, unlike GeneXpert. I will also remind them that the battery is not detachable, you cannot remove the battery, if you want to charge the battery, you have to carry the entire machine along but if the battery. If I the battery is detachable, I can leave the machine in my village to go and charge the battery and bring it back, since I know that it is like that, I will tell them to find a way to make the battery detachable so that it will be easy for us to work around it. These are the key things, I will also remind them that GeneXpert machines, you will do your sample for instance, in one hour or about that, you get MTB result, you will get RIF result at the same time. With Truenat, you need to do your analysis for MTB first, when you finish all the runs, you come back to process sample and start testing for RIF resistance. So, at the end of the day, there is an impact on that on quantification because you might quantify for MTB and RIF the same time, what of if you do 50 tests and all the 50 are MTB and there is no RIF, what happens to the reagent you have quantified that is already in country, and we have already placed a cost on it. So, these are some of the elements that we need to look at and say, if we see them and sit down with them, we can discuss, and I know that they will improve on it. |
| Thank you very much Sir. | Thank you too |
