# Supplementary material for: Exploring the perspectives of healthcare workers and Program managers on the use of Truenat as a new tool for TB and DR-TB diagnosis in Nigeria: A qualitative study
Source: PLoS One. 2024 Dec 30;19(12):e0316204. doi: 10.1371/journal.pone.0316204 (PMC11684725; doi:10.1371/journal.pone.0316204)
Supplement: Attachment — Submitted filename: PONE-D-24-17897_review-rvsd-08-25-2024.pdf [file pone.0316204.s004.pdf]

Background

1. The gap in knowledge is not adequately captured in the background.
2. What is the contribution to the body of science that this study aims to provide?
3. Some background on the TB-LON1,2 & 3 projects and the KNCV TB foundation should be included in the background. For instance, readers need to know what the main difference is between TB-LON 1&2 and TB LON-3. – Not addressed
4. What do we know in literature about the functionality and promises that Truenat offers, especially in other countries where this innovation has been implemented? Authors need to provide some information on this. Satisfactorily addressed
5. Line 77. There has been significant global research…… Please provide a citation to global research to substantiate the claim. Not addressed
6. The sentence on Line 104: "Nigeria is one the ten countries that contributes……….Dr-TB and HIV associated TB" is better moved to Line 71 where missing cases were first mentioned right after "……are from developing countries". In this way, a flow is enhanced for the argument on "missing cases" and also the objectives of the study are not interrupted as it currently is on Line 104. Not addressed
7. When the sentence on Line 104 "Nigeria is one the ten countries that contributes……….Dr-TB and HIV associated TB" , has been taken off. Authors should revise the next sentence on objectives to include "**also**", i.e., "This study was **also** designed to assess the enablers and barriers……in Nigeria". (The reason why "also" is suggested here is because an there is a prior objective stated on Line 102 above.) Not addressed

Methods

8. Line 118. Why is the "Southern part of the country" in Caps? Southern should be southern (Not capitalized). The context in which Southern is used in that sentence is not a noun. Only nouns can have their first letters capitalized when the word is used within a sentence. Authors need to submit the manuscript to an English reviewer to ensure that these minor grammatical issues can be corrected. Not addressed
9. It is also important for authors to describe what form of analysis was conducted – Was the analysis conducted using inductive or deductive analysis. And this would directly extend into the type of coding that was used to analyze the themes and sub-themes that were generated from the transcripts (data). Addressed
10. A tabular representation of the participants, highlighting the gender, age, roles of each of the participants would be useful for readers to understand some assertions in the quotes/analyzed results. Not addressed
11. Authors have not described how the biases in qualitative research have been addressed or minimized particularly the Reflexivity, Selection, Response, Participant, Common method and Non-Response Biases. Addressed
12. On line 145, pre-test was conducted among how many participants? Please specify if pre-test was conducted specifically for both FGD and KII guides or not. Addressed
13. On line 152, authors state that an average duration **of the FGDs** was one hour twenty minutes……
    a. How many FGDS were conducted?
    b. How many participants were on each FGD?

   c. Is the time mentioned here the time for each of the FGDs or the total time for all the FGDs together? Authors please clarify. Not addressed

14. The same issues apply to Line 153 on KIIs "......while for **the KII**, it was fifty-five minutes"
   a. The use of KII in this sentence presupposes that only one key informant interview was done instead of three in number – since the KII is written in singular. Again, if this is a typo, these are the issues that require an English editor to address as recommended above.
   b. Did one Key Informant Interview last for 55minutes for each interview or what the 55minutes the total time for the 3 program managers altogether. Authors please clarify.
15. On Line 154, authors state that the transcripts of the interviews were returned to the participants for comments. What comments were the authors seeking for that the interviews didn't adequately capture already. The purpose of the qualitative interviews is to generate as much data during the interview. Can authors kindly justify or provide the importance of this activity please?
16. On line 157, what language was used to conduct the interviews – English? Authors should kindly add this information to the manuscript. Authors should describe how the quality of the data was ensured if the language utilised was conducted in any other language other than English. Addressed
17. On Line 163, "The themes from each interview were reviewed **by the** researchers....". Who are the researchers? Are the researchers the same as the reviewers on line 158 & 161. Authors should clarify this please. Addressed
18. How were the researchers selected – based on what qualifications/characteristics were the researchers selected? The importance of this cannot be over-emphasized as it helps to improve the data quality and the validation of the results presented. Addressed
19. On line 164. Authors should kindly provide a reference for Nvivo statistical software - Not addressed

Results

20. What does (SQAO) on line 230 stand for? Even though it is listed in the abbreviations section, it should be written out in its 1st use within the manuscript. Addressed
21. Authors need to be consistent in the reporting of results. In some cases, participants were addressed by their roles (Clinician, Laboratory Staff) while in other quotes, KII participant was used. It is recommended that authors use a consistent method to capture the owners of the quotes in the results for all FGDs and KIIs. A format that can be adopted is provided here - (KII-3, Male,44years, Clinician) Addressed
22. On line 487, "A participant has an impression...." This should be in past tense since the results are in reported speech and so that the past tense used for reported speeches can match uniformly with the tense in the methods and results sections. Not addressed
23. On line 488, please close the gap between "the" and "GeneXpert". Not addressed
24. On line 523, "however issues related...", should include a comma, – "However, issues related". A number of grammatical issues need to be addressed in this section as well Not addressed.
25. On line 733. Conclusions should not be plural. Not addressed

New Query

26. On Line 200 and 479: What does 3 D U W L F L S D Q W V S U R I L O H stand for?

Note to Authors

Some of the concerns raised have not been fully addressed. The yellow highlights on the re-submitted corrected version is good. Authors should kindly endeavor to refer to all queries that are un-addressed and provide a correction, a refute or an explanation for the points that remain unaddressed.
