# Supplementary material for: Exploring the perspectives of healthcare workers and Program managers on the use of Truenat as a new tool for TB and DR-TB diagnosis in Nigeria: A qualitative study
Source: PLoS One. 2024 Dec 30;19(12):e0316204. doi: 10.1371/journal.pone.0316204 (PMC11684725; doi:10.1371/journal.pone.0316204)
Supplement: Attachment — Submitted filename: RESPONSE.docx [file pone.0316204.s003.docx]

**Review comments and response from Authors 2**

**Comment**

We note that this data set consists of interview transcripts. Can you please confirm that all participants gave consent for interview transcript to be published?

**Author response**

**I confirm that all the participants consented for the interview transcripts to be published**

**Comment**

If they DID provide consent for these transcripts to be published, please also confirm that the transcripts do not contain any potentially identifying information (or let us know if the participants consented to having their personal details published and made publicly available). We consider the following details to be identifying information:

**Author response**

**Thanks for the observation. The participants DID provide consent for these transcripts to be published. The interview transcripts have been reviewed again and all potentially identifying information have been removed.**

**Comment**

We note that your Data Availability Statement is currently as follows: "All relevant data are within the manuscript and its Supporting Information files"

**Author response**

**All relevant data related to the manuscript is uploaded as a Supporting Information file named ‘Transcripts.’**
